# Metal Transporter Gene *SLC39A8* Polymorphism rs13107325 and Dietary Manganese Intake Are Associated with Measures of Cardiovascular Disease Risk in a UK Biobank Population Cohort

**DOI:** 10.3390/nu17193031

**Published:** 2025-09-23

**Authors:** Riju Sigdel, Parker R. Johnson, Gracie E. Meade, Aiden Y. Kim, Gracie M. Maschmeier, Edralin A. Lucas, McKale R. Montgomery, Dingbo Lin, Sam R. Emerson, Winyoo Chowanadisai

**Affiliations:** 1 Department of Nutritional Sciences, Oklahoma State University, 301 Nancy Randolph Davis Building, Stillwater, OK 74078, USA; riju.sigdel@okstate.edu (R.S.); parjohn@okstate.edu (P.R.J.); gracie.meade@okstate.edu (G.E.M.); yejoon.kim@okstate.edu (A.Y.K.); gracie.maschmeier@okstate.edu (G.M.M.); edralin.a.lucas@okstate.edu (E.A.L.); mckale.montgomery@tcu.edu (M.R.M.); dingbo.lin@okstate.edu (D.L.); sam.emerson@okstate.edu (S.R.E.); 2 Department of Nutritional Sciences, Texas Christian University, Fort Worth, TX 76109, USA

**Keywords:** ZIP8, heart disease, obesity, BMI, nutrigenomics, genetics

## Abstract

**Background/Objectives:** Metal transporter gene *SLC39A8* and single nucleotide polymorphism (SNP) rs13107325 are associated with risk factors for atherosclerosis and cardiovascular disease (CVD). However, it is unclear how dietary manganese intake impacts CVD risk factors. The aim of this study was to use the UK Biobank population cohort (276,436 participants, Caucasian genetic ancestry, no genetic kinship) to investigate whether rs13107325 and dietary manganese are associated with CVD risk. **Methods:** A cross-sectional design and quantile (median) regression was used to determine associations of rs13107325 and dietary manganese intake with indicators of CVD risk. **Results:** SNP rs13107325 was associated with CVD risk factors, including greater body mass index (BMI) (beta ± SE per rs13107325 allele = 0.283 ± 0.0392, false discovery rate (FDR) < 10^−10^) and triglycerides (beta ± SE = 0.0308 ± 0.00761, FDR < 0.001) and reduced high density lipoprotein (HDL) (beta ± SE = −0.0298 ± 0.00343, FDR < 10^−15^). SNP rs13107325 was also associated with lower systolic (beta ± SE = −0.601 ± 0.172, FDR < 10^−3^) and diastolic blood pressure (beta ± SE = −0.531 ± 0.100, FDR < 10^−5^). Dietary manganese intake was positively correlated with measures of favorable cardiovascular health, such as lower BMI (beta ± SE per mg dietary manganese = −0.531 ± 0.0118, FDR < 10^−300^), reduced triglycerides (beta ± SE = −0.0451 ± 0.00229, FDR < 10^−50^), increased HDL (beta ± SE = 0.00958 ± 0.00103, FDR < 10^−15^), and lower blood pressure (systolic beta ± SE = −0.529 ± 0.0520, FDR < 10^−20^; diastolic beta ± SE = −0.562 ± 0.0302, FDR < 10^−50^). **Conclusions:** The favorable associations of dietary manganese opposed many deleterious trends associated with rs13107325. Increased dietary manganese may promote cardiovascular health and offset many risks to cardiovascular health linked to SNP rs13107325.

## 1. Introduction

Many cardiovascular diseases (CVD) are chronic and progressive, eventually manifesting in life-threatening and often fatal events such as myocardial infarction [1]. Atherosclerosis is a key mechanism in CVD accelerated by obesity and dyslipidemia [1]. Aging is also associated with reduced muscle strength and sarcopenic obesity, which can contribute to atherosclerosis and CVD [2]. Although macronutrients such as refined carbohydrates and saturated fats are well-recognized dietary risk factors of atherosclerosis [3], there is less knowledge regarding the importance of trace minerals like manganese and their impacts on CVD risk.

The gene *SLC39A8* encodes a metal transporter, ZIP8, that is permeable to manganese, zinc, and iron [4,5]. Homozygous, loss-of-function mutations in the *SLC39A8* gene lead to severe manganese deficiency, with modest bearings on iron and zinc metabolism, and causes a congenital disorder of glycosylation [6,7]. Clinical symptoms in this disease can be improved by high doses of oral manganese supplementation [8]. Similarly, global and tissue-specific knockout of ZIP8 in mice leads to indications of manganese deficiency including reduced blood or tissue manganese concentrations [9,10,11,12]. Biochemically, manganese serves as a cofactor for several enzymes, such as manganese superoxide dismutase, arginase, and glycosyltransferases [13]. Collectively, these manganese-dependent metalloenzymes impact antioxidant protection, cellular metabolism, and protein glycosylation [12,13]

SNP rs13107325 results in a missense mutation with reduced cell surface accumulation of ZIP8 and lower manganese transport ability in cellular and radiotracer studies [9,14,15]. People with rs13107325 exhibit low blood and plasma manganese [14,16], which may be attributed to reduced intestinal manganese absorption [9] and impaired reclamation of manganese from bile [11,12]. Lower manganese-dependent galactosyltransferase activity [11] due to rs13107325 causes secondary hypoglycosylation [8] and affects physiological systems important for cardiovascular function. SNP rs13107325 is highly represented in populations of Northern European or Ashkenazi Jewish origins, with an estimated 14% and 25–37% of these populations carrying one risk allele, respectively [17]. Polymorphism rs13107325 is considered the top candidate and causative SNP in genome-wide association studies (GWAS) within the *SLC39A8* locus for greater BMI [18], reduced HDL [19], and higher triglycerides [20]. SNP rs13107325 was also associated with lower blood pressure in a previous GWAS [21]. SNP rs13107325 is associated with elevated plasma *N*-terminal prohormone of brain natriuretic peptide (NT-proBNP) levels and greater risk of death from CVD [22]. Dietary manganese is associated with lower CVD prevalence [23] and mortality [24]. Thus, it is plausible that dietary manganese intake may increase manganese status and therefore partially offset some of the negative impacts of rs13107325 associated with obesity and cardiovascular disease.

The UK Biobank is a population cohort that consists of individuals age 40–69 years at enrollment [25,26] and provides an opportunity to study genetic and health factors in a human population that is middle to late aged. The UK Biobank contains lifestyle and health measures relevant to CVD, including estimated dietary nutrient intake data for an expanded range of micronutrients such as manganese [27]. The recent inclusion of micronutrient intake information now enables the combined study of dietary manganese and *SLC39A8* SNP rs13107325 to determine their associations with risk factors for CVD. We hypothesized that greater dietary manganese intake would be associated with a more optimal cardiometabolic risk profile, such as less adiposity and favorable blood lipid profiles, and offset or ameliorate deficits caused by rs13107325. We also hypothesized that higher dietary manganese intake and rs13107325 would be associated with lower blood pressure.

## 2. Methods

### 2.1. UK Biobank Baseline Characteristics

Adult participants from the UK Biobank included over 500,000 males and females aged between 40 and 69 years old in the United Kingdom and were recruited between 2006 and 2010 [28]. Subjects completed questionnaires and verbal interviews covering sociodemographic, lifestyle, dietary, and disease histories as well as physical assessments and non-fasted blood sampling [28]. Baseline characteristics are provided (Table 1). Additional imaging assessments were conducted, and follow-up dietary recalls were performed. This data was used to perform observational analyses using a cross-sectional design.

We filtered the UK Biobank cohort (dataset dispensed 17 January 2024) to include subjects of Caucasian genetic ancestral grouping and without genetic kinship to other participants because rs13107325 is concentrated in this population and mostly absent in other groups [17] and to avoid familial confounding. Use of genetic ancestry provides a more defined population compared to self-identified race or ethnicity categories [29]. Genetic kinship was estimated from pairwise kinship coefficients, and third-degree relatedness qualified as kinship [26]. These two filters reduced the cohort from 502,233 to 276,436 participants (Supplemental Figure S1). The final cohort is fully 100% Caucasian self-reported ethnicity and 100% Caucasian genetic ancestry.

The UK Biobank study was conducted under the guidance of the North West Multi-Centre Research Ethics Committee (reference number: 06/MRE08/65). This project was determined by the Oklahoma State University Institutional Review Board to be Not Human Subjects Research (IRB-21-432, 12 October 2021). Variables used are listed by UK Biobank variable identification numbers in Supplemental Table S1.

### 2.2. SLC39A8 SNP rs13107325 Genotype (Predictor)

Genotypes were previously measured by the UK Biobank Axiom Array and imputed [26]. SNP rs13107325 is present on the Axiom array. Genotype data was obtained from bgen files on the UK Biobank Research Access Platform (RAP) and converted to vcf format using *plink2*, *bcftools*, and *vcftools* [30,31,32] through the UK Biobank RAP Swiss Army Knife app. For each subject, SNP rs13107325 genotype status was coded as an additive model with each person possessing 0, 1, or 2 risk alleles.

### 2.3. Manganese Intake (Predictor) from 24 h Dietary Recalls

The UK Biobank contains daily dietary manganese intake data through 24 h dietary recalls, which was recorded once in-person and four subsequent online surveys. The Oxford WebQ tool for nutritional assessment was used for deriving nutrient data from 24 h dietary recalls with updated calculations to include manganese [27]. Multiple pairwise correlations show that each manganese record was evenly correlated with the other manganese datapoints for each individual (all 5 records: r = 0.454–0.524, N = 3398). Assigning manganese intake as a variable with at least one entry out of 5 total (multiple entries averaged) allows for maximal sample size. If sample size is sufficient for certain outcomes, we used average intake among the limited individuals (N = 16,057) with all 4 online records (ignoring in-person data), which provided the most consistent representation of dietary manganese intake (r = 0.847–0.858 between averaged intake and each separate online record, instances 1–4). A major loss of sample size (from 16,057 down to 3398) occurred if analyses required subjects with all 5 dietary entries. Because some foods are rich in multiple minerals or fiber, the consideration of other nutrients allowed us to determine the specificity of observed associations to manganese in case they could be attributed to other nutrients. Energy intake was included as a general measure of food consumption [33]. Bioavailable iron was estimated from elemental iron absorption efficiency at 10% and heme iron at 25% [34]. Dietary intakes for zinc and selenium were also obtained. Fiber, categorized as non-starch polysaccharides represented as Englyst dietary fiber, is rich in some vegetables and legumes also high in manganese. Dietary records (instances) that were flagged as “Daily dietary data not credible” were omitted. To confirm that our findings were not confounded by unusual dietary behavior among participants, we performed a separate analysis omitting dietary records (instances) that were self-reported as a dietary intake not typical for that person that day. We examined if our findings were still significant when predominantly evaluating food sources of manganese by excluding dietary recalls with documented vitamin or mineral supplement use for that period in a subset of participants.

### 2.4. Primary and Secondary Outcomes

Primary outcomes for this study included a collection of measurements indicative of adiposity (BMI) and metabolic health (HDL, triglycerides) and associated with rs13107325 in previous GWAS [18,19,20]. We hypothesized that rs13107325 and dietary manganese would have opposing associations for these three health measures.

The secondary outcome in this study was blood pressure (systolic and diastolic blood pressure). Two automated measures of blood pressure were taken a few moments apart using Omron 705 IT electronic blood pressure monitor (Omron Corporation, Kyoto, Japan). The average of the two automated readings was used in this study.

### 2.5. Phenotype Data (Including Primary Outcomes and Additional Covariates)

Phenotype data was downloaded from the UK Biobank RAP through the Table Exporter app version 2.0.78. For blood-based biomarkers, fasting time (i.e., time since last meal) was included as a covariate given that blood collection was performed under random (non-fasted) conditions [35]. Methods for plasma nuclear magnetic resonance (NMR) metabolomics [36], dual x-ray absorptiometry (DEXA) [37], and magnetic resonance imaging (MRI) [37] are described elsewhere. Education level was accounted through subject’s achieved certifications, which were mapped to different levels of the International Standard Classification of Education (ISCED) of the United Nations Educational, Scientific and Cultural Organization [38]. Household income was accounted using average total income before tax received by a household. Townsend deprivation index is a general measure of socioeconomic status based upon the neighborhood of the enrollment center from which subjects are recruited. Use of cholesterol-lowering medications, antihypertensive medications, insulin, or aspirin were noted. Physical activity was accounted for, using metabolic equivalent task (MET) minutes per week (<1000, 1000 to <2000, 2000 to <4000, ≥4000). MET was considered as walking, moderate and vigorous activity derived from Oxford WebQ questionnaire. Additional covariates include age, sex, tobacco smoking status at enrollment, provided as current user, previous user, or never smoked, and alcohol intake frequency at enrollment (daily or almost daily, 3 or 4 times/week, 1 or 2 times/week, 1 to 3 times/month or special occasions only). Genotype and phenotype data were merged by pseudo-anonymized participant ID. Missing data or subject non-responses (“Prefer not to answer”, “Do not know”, or “None of the above”) were coded as blank entries.

### 2.6. Population Stratification (Covariate) Measures Through Genetic Principal Components

Observed SNP associations may unintentionally reflect other genetic differences co-segregating with rs13107325, the SNP of interest. Failure to account for population stratification, even within the Caucasian genetic ancestral group, can confound scientific data interpretation [39]. To account for population stratification, principal component markers of genetic variance were added as covariates through 40 provided eigenvectors derived by *shellfish* version 1.0 software [26].

### 2.7. Statistics

Associations between predictors and continuous outcomes were analyzed by quantile (median) regression as a nonparametric approach. Significance was set at *p* < 0.05. Stata (versions 15.1, 17, and 18) and R version 4.5.0 were used for statistical analyses. We corrected for multiple comparisons using the Benjamini–Hochberg method to yield the false discovery rate (FDR). Significance was set at FDR < 0.05.

## 3. Results

### 3.1. Associations of rs13107325 and Dietary Manganese Intake with Health Measures

Similarly to findings in previously reported studies [18,19,20], we found that *SLC39A8* SNP rs13107325 is associated with greater BMI, lower HDL, and higher triglycerides (Table 2). We found that dietary manganese is associated with lower BMI, greater HDL, and lower triglycerides (Table 2 and Table 3). These associations were linear across the range of dietary manganese intakes in the cohort. These findings were present and largely unchanged after the inclusion of genetic principal components as measures of population stratification (Table 2 and Table 3).

We performed additional analyses to ensure that our observed relationships were consistent even when considering other factors such as dietary report consistency or use of dietary supplements. Associations between dietary manganese and BMI, HDL, and triglycerides remained significant when examining a smaller cohort with multiple dietary records per subject (Supplemental Table S2). Associations between rs13107325 and HDL were also significant in the smaller cohort with multiple dietary records per subject (Supplemental Table S2). The beta regression coefficients for rs13107325 and the other measures in this smaller cohort (Supplemental Table S2) were similar to those in the larger cohort with at least one dietary entry (Table 2 and Table 3) but failed to reach significance due to greatly reduced sample sizes. Associations for rs13107325, dietary manganese, and these CVD risk factors were retained after analyzing only individuals who reported that their dietary recall reflected their typical diets (Supplemental Table S3), and there were no observable differences when excluding self-reported atypical dietary data. Associations for rs13107325, dietary manganese, and these measures of CVD risk were also significant when analyzing dietary recalls without multivitamin supplement use (Supplemental Table S4). In summary, associations for rs13107325, dietary manganese, and these health measures were largely consistent and not unduly impacted by subject consistency of reporting dietary recalls or use of multivitamins.

To account for possible dietary confounders, particularly plant-derived food sources with micronutrient content overlapping with manganese, we included fiber, iron, zinc, and selenium in another regression model. For BMI, HDL, and triglycerides, the associations with rs13107325 and dietary manganese remained robust (Supplemental Table S5). In general, associations were still significant even when accounting for additional trace minerals and fiber as covariates.

We considered possible interactions between rs13107325 and dietary manganese in our regression analyses for CVD risk factors. With the possible exception for triglycerides in females (interaction trend *p* = 0.0604), no significant interactions were found between rs13107325 and dietary manganese (as main effects) and their associations with these CVD risk factors (Supplemental Table S6).

To uncover possible sex differences, we compared effect sizes of rs13107325 and dietary manganese intake on outcomes between males and females in sex-stratified analyses (Table 2 and Table 3). Effect sizes, as observed as betas in regression analyses, were largely similar between males and females. However, males showed reduced effect size and greater variability with rs13107325 and circulating triglycerides compared to females. For serum HDL, males had reduced effect sizes due to both rs13107325 and dietary manganese intake.

### 3.2. Association of rs13107325 and Dietary Manganese with Overall Adiposity

We further examined the association between *SLC39A8* SNP rs13107325 and BMI by analyzing other measures of adiposity. We found that rs13107325 is associated with greater body fat percentage for both males and females when examining body composition measured by impedance (Table 4 and Table 5, Supplemental Table S7). However, relative to body fat percentage, the association between rs13107325 and waist circumference was less pronounced in males and not significant in females. Dietary manganese intake was highly associated with lower body fat percentage and waist circumference. Associations between rs13107325 and measures for visceral adiposity, such as trunk fat percentage, paralleled associations with whole body fat percentage. Elevated whole body, arm and leg relative fat composition indicate an overall increase in adiposity associated with rs13107325 and a similar breadth of reduction in adipose correlated with dietary manganese. Dietary manganese was also associated with lower fat-free or lean mass, but generally to a lower extent than fat mass. SNP rs13107325 was associated with fat-free mass in females but not males. Results from both dual-energy X-ray absorptiometry (DEXA) and abdominal magnetic resonance imaging (MRI) (Supplemental Table S8) generally paralleled body impedance data regarding dietary manganese and lower adiposity. There was insufficient sample size for representation of rs13107325 risk alleles in subjects with DEXA or MRI data given the need for an imaging visit.

### 3.3. Association of rs13107325 and Dietary Manganese with Lipid and Lipoprotein Metabolism

We examined associations between *SLC39A8* SNP rs13107325 and lipoproteins and lipid metabolomics. The negative association between rs13107325 and HDL was also present for apolipoprotein A (ApoA) (Table 6 and Table 7), whereas negative association between rs13107325 and low density lipoprotein (LDL) was less robust and there was no significant association found between rs13107325 and apolipoprotein B (ApoB) (Table 6 and Table 7). Dietary manganese was associated with higher lipoprotein A and lower *C*-reactive protein (CRP) (Table 6 and Table 7), whereas rs13107325 was not associated with these two biomarkers. For a subset for which subject blood samples were analyzed by NMR metabolomics, we examined lipoprotein characteristics for associations with rs13107325 and dietary manganese. Lower HDL concentrations associated with SNP rs13107325 likely reflect reduced reverse cholesterol transport ability, which is supported by the lower amount of cholesterol present in medium, large and very large HDL subfractions (Table 8) [40]. The reduced LDL size linked to rs13107325 likely reflects greater atherogenic potential (Table 8) [41]. Larger VLDL size associated with rs13107325 is consistent with elevated circulating triglycerides and may be a contributor to the small, dense LDL (Table 8) [42]. The high amounts of triglycerides present in HDL and LDL potentially reflect dysfunctional HDL with impaired reverse cholesterol transport function and greater atherogenicity from LDL, respectively (Table 8) [43,44]. For most lipid parameters, dietary manganese intake exhibited associations that were countertrend to rs13107325 and consistent with more optimal lipid metabolism, such as larger HDL and LDL particles, greater HDL concentration, and reduced triglyceride content in HDL and LDL (Table 8). These associations for dietary manganese are also present and remain significant for the smaller cohort with multiple dietary records per subject (Supplemental Table S10).

### 3.4. Association of rs13107325 and Dietary Manganese with Blood Pressure

SNP rs13107325 was associated with reduced systolic and diastolic blood pressure in the mixed cohort with both sexes (Table 9 and Table 10). However, sex-specific analyses showed that decreases in systolic blood pressure associated with rs13107325 are more pronounced in females, whereas only a non-significant trend was present in males (*p* = 0.0715) (Table 9). Contrary to differing results between rs13107325 and dietary manganese for adiposity and lipid metabolism, both rs13107325 and dietary manganese were associated with reduced systolic and diastolic blood pressure (Table 9 and Table 10).

## 4. Discussion

The present study analyzed *SLC39A8* SNP rs13107325 and dietary manganese in the context of cardiovascular health. Though previous studies have observed associations between higher dietary manganese intake and elevated serum manganese levels with reduced cardiovascular disease outcomes in general population cohort [23,24,45,46], these associations with cardiovascular health have not been previously extended to people with *SLC39A8* rs13107325 polymorphisms. In patients with severe *SLC39A8* mutations, high dose of dietary manganese supplementation improved clinical outcomes and corrected biochemical deficits [8]. The novel results in this study show that higher intakes of manganese, within normal food consumption patterns, are associated with improved cardiovascular health measures. Thus, our findings demonstrate the potential for precision nutrition interventions aimed at optimizing dietary manganese to improve cardiovascular health in populations with *SLC39A8* rs13107325 risk alleles. We found that rs13107325 and dietary manganese have largely independent and opposing associations with multiple measures correlated with cardiovascular health, which would imply that greater manganese intake may counteract the effects of rs13107325. Thus, our findings demonstrate the clinical potential for precision nutrition interventions aimed at optimizing dietary manganese to improve cardiovascular health in populations with *SLC39A8* rs13107325 risk alleles. SNP rs13107325 appears to affect adipose distribution broadly, and dietary manganese intake may counter adiposity.

Previous studies have linked rs13107325 to higher circulating NT-proBNP and von Willebrand factor (VWF) levels, which are associated with cardiovascular disease risk. In a previous GWAS, Johansson et al. [22] reported that in acute coronary syndrome patients, rs13107325 was associated with elevated plasma NT-proBNP levels and higher risk of cardiovascular death. Levels of NT-proBNP are positively associated with a higher risk of ischemic and hemorrhagic strokes [47]. In a GWAS examining plasma coagulation factors concentrations, Sabater-Lleal et al. [48] identified *SLC39A8* as a possible locus for VWF levels and found elevated VWF release from human endothelial cells depleted of ZIP8 (*SLC39A8*) by siRNA transfection. Our regression models indicate that for BMI, approximately 0.531 mg more dietary manganese intake per day would offset the 0.283 unit increase in BMI associated with each rs13107325 risk allele. In contrast, both rs13107325 and dietary manganese are associated with lower blood pressure. About 90% of our UK Biobank cohort surpass the adequate intake (AI) for manganese, yet our findings show that dietary manganese consumption above the AI is associated with improved measures of cardiovascular health.

We found that rs13107325 affects adiposity across the body and alters lipid metabolism. Disruptions in lipoprotein metabolism associated with rs13107325 are consistent with impaired reverse cholesterol transport and increased atherogenic potential, as evidenced by shifts in lipoprotein particle sizes and cholesterol to triglyceride compositions among lipoprotein subclasses. Importantly, greater manganese intake was associated with enhanced cardiovascular measures that ran countertrend to rs13107325 and appeared to offset this deleterious risk polymorphism.

Our data suggests that rs13107325 broadly affects overall adiposity, as observed by higher BMI and body fat percentage, without selectively impacting visceral adiposity in both males and females. In addition, we did not observe any links between rs13107325 and systemic inflammation as analyzed by *C*-reactive protein. One interpretation could be that the lack of association between rs13107325 and systemic inflammation suggests that other factors other than inflammation play a role in how rs13107325 and ZIP8 affect cardiovascular health and function. In contrast, dietary manganese has a robust negative association with measures of adiposity, including waist circumference and trunk fat, and *C*-reactive protein as an indicator of inflammation.

It should be noted that other studies have observed links between ZIP8, rs13107325, and immune response. In knock-in mice modeled after the rs13107325 polymorphism, both Nakata et al. [10] and Yang et al. [49] found signs of intestinal inflammation that parallel previously reported associations in humans between rs13107325 and Crohn’s disease [50,51]. Overexpression of human ZIP8 containing the rs13107325 variant resulted in greater TNF-α-induced NF-κβ activation relative to the reference version [52], which is consistent with an increased inflammatory response to pro-inflammatory cytokine activation. Hypomorphic ZIP8 mice have impaired host defense, increased NF-κβ activation, and excessive inflammation during experimentally induced sepsis [53]. Computational modeling of gene expression in monocytes supports a role for ZIP8 in atherosclerosis that is partially mediated by HDL, additionally induced by plaque formation independent of HDL, and also influenced by environmental (and often carcinogenic) factors such as cadmium exposure, tobacco smoking, and TNF-α induced inflammation [54]. In cadmium-exposed rat lung epithelial cells, ZIP8 silencing by epigenetic hypermethylation downregulates ZIP8 expression [55]. Overall, ZIP8 has been implicated in inflammation in other diseases. Accordingly, the shared pathogenic mechanisms between inflammatory bowel diseases, pulmonary fibrosis, lung carcinogenesis, and atherosclerosis [56]. Consequently, another possibility is that ZIP8 and rs13107325 may still affect pro-inflammatory processes involved in cardiovascular disease even without elevated *C*-reactive protein concentrations.

Given the observational nature of this UK Biobank cohort study, the interpretation of these findings has limitations that are common to many biobank-based associational studies. Where possible, we have attempted to consider whether other trace elements, such as iron, zinc, and selenium, instead of dietary manganese may affect the observed associations. This study investigated dietary manganese intake but measures of manganese status, such as blood, serum, or plasma manganese concentrations, are not available in the UK Biobank dataset. Previous studies investigating manganese metabolism have shown that rs13107325 is associated with lower blood and plasma manganese concentrations [11,14,16]. The lack of most micronutrient status indicators in the UK Biobank is understandable given that this cohort was designed to maximize subject participation and data for the widest range of scientific questions within budget constraints [35]. One significant constraint for the UK Biobank is that only 10 percent of this ongoing cohort (24,715 out of 276,436) is deceased. Accordingly, the existing UK Biobank data for analyzing CVD incidence and mortality associated with rs13107325 and dietary manganese is currently insufficient but should become adequately powered in the future. Future investigations should consider other possible conditions with interactions between rs13107325 and dietary manganese.

## 5. Conclusions

SNP rs13107325 and dietary manganese show opposing associations with adiposity and cardiometabolic measures associated with CVD. Further studies are warranted to determine if CVD can be ameliorated by increases in manganese intake from foods or modest dietary supplementation while balancing the negative potential of excess manganese exposure.

## Figures and Tables

**Table 1:** Baseline characteristics of UK Biobank population cohort.

Characteristics	Females	Males
Number of subjects (N)	147,348	129,088
SNP rs13107325 risk alleles (percent of sex-specific cohort)	0: 126,378 (85.8%)	0: 110,204 (85.4%)
	1: 20,191 (13.7%)	1: 18,189 (14.1%)
	2: 779 (0.529%)	2: 695 (0.539%)
Age at recruitment (years)	58 (50, 63)	59 (51, 64)
Height (cm)	163 (159, 167)	176 (171.8, 180.7)
Weight (kg)	69.1 (61.8, 78.6)	84.6 (76.5, 94)
Dietary manganese intake (mg/day)	4.00 (3.15, 4.91)	4.23 (3.30, 5.30)
	N = 65,466	N = 56,133
Dietary energy intake (kJ/day)	7863 (6649, 9192)	9157 (7703, 10785)
	N = 65,466	N = 56,133
Townsend deprivation index	−2.38 (−3.76, −0.03)	−2.37 (−3.78, 0.11)
Household income (GBP):	Percentage (N):	Percentage (N):
Less than 18,000	23.1% (28,307)	18.9% (21,990)
18,000–30,999	26.5% (32,463)	24.4% (28,400)
31,000–51,999	25.8% (31,644)	27.3% (31,737)
52,000–100,000	19.5% (23,913)	23.1% (26,786)
Greater than 100,000	5.03% (6163)	6.27% (7285)
Qualifications (education):	Percentage (N):	Percentage (N):
None of the listed options (lowest)	16.3% (23,838)	16.3% (20,874)
CES or equivalent, O levels and GCSEs or equivalent	20.0% (29,171)	13.7% (17,479)
A levels/AS levels or equivalent	5.88% (8595)	5.1% (6551)
Other professional qualifications (such as nursing or teaching)	13.9% (20,319)	10.9% (13,989)
College/university degree, NVQ/HND/HNC or equivalent (highest)	43.9% (64,208)	54.0% (69,052)
Smoking:	Percentage (N):	Percentage (N):
Never smoked	59.7% (87,651)	49.4% (63,513)
Previous smoker	31.8% (46,735)	38.9% (50,006)
Current smoker	8.5% (12,477)	11.8% (15,113)
Alcohol intake:	Percentage (N):	Percentage (N):
Never	7.8% (11,442)	4.9% (6290)
Special occasions only	13.7% (20,221)	6.5% (8404)
One to three times a month	13.0% (19,095)	8.8% (11,293)
Once or twice a week	26.2% (38,554)	25.9% (33,427)
Three or four times a week	21.9% (32,210)	27.0% (34,880)
Daily or almost daily	17.5% (25,720)	26.9% (34,704)
Medication use (antihypertensive medications, cholesterol-lowering medications, aspirin, and insulin):	Percentage (N):	Percentage (N):
No medication use	73.9% (108,785)	62.9% (81,062)
One of four medications	15.9% (23,412)	16.8% (21,655)
Two of four medications	6.8% (10,068)	11.2% (14,443)
Three of four medications	3.1% (4571)	8.6% (11,104)
All four medications	0.3% (368)	0.5% (697)
Summed Metabolic Equivalent Task (MET) minutes per week for all activities:	Percentage (N):	Percentage (N):
Less than 1000 min per week	22.7% (33,504)	24.7% (31,931)
1000 to 1999 min per week	18.2% (26,747)	19.1% (24,616)
2000 to 3999 min per week	18.5% (27,224)	19.9% (25,645)
Greater or equal to 4000 min per week	40.6% (59,873)	36.3% (46,896)

Distributions are presented as median (25th percentile, 75th percentile). Numbers (N) are provided when data was available for less than 99 percent of the subjects.

**Table 2:** Association of SLC39A8 SNP rs13107325 with BMI, circulating HDL and triglycerides.

	Model	Both Sexes			Female Only			Male Only		
		N	rs13107325 (Beta ± SE)	FDR rs13107325	N	rs13107325 (Beta ± SE)	FDR rs13107325	N	rs13107325 (Beta ± SE)	FDR rs13107325
BMI	Model 1	121,236	0.297 ± 0.0376	<10^−10^	65,275	0.291 ± 0.0563	<10^−5^	55,961	0.266 ± 0.0504	<10^−5^
	Model 2	108,691	0.283 ± 0.0392	<10^−10^	56,777	0.242 ± 0.0586	<10^−3^	51,914	0.292 ± 0.0494	<10^−5^
	Model 3	108,691	0.271 ± 0.0384	<10^−10^	56,777	0.258 ± 0.0591	<10^−3^	51,914	0.293 ± 0.0503	<10^−5^
HDL (mmol/L)	Model 1	105,853	−0.0319 ± 0.00354	<10^−15^	56,519	−0.0400 ± 0.00545	<10^−10^	49,334	−0.0262 ± 0.00448	<10^−5^
	Model 2	94,896	−0.0298 ± 0.00343	<10^−15^	49,134	−0.0382 ± 0.00515	<10^−10^	45,762	−0.0242 ± 0.00429	<10^−5^
	Model 3	94,896	−0.0300 ± 0.00345	<10^−15^	49,134	−0.0364 ± 0.00513	<10^−10^	45,762	−0.0231 ± 0.00436	<10^−5^
Triglycerides (mmol/L)	Model 1	115,691	0.0326 ± 0.00763	<10^−5^	62,282	0.0366 ± 0.00814	<10^−5^	53,409	0.0390 ± 0.0131	0.00293
	Model 2	103,704	0.0308 ± 0.00761	<10^−3^	54,171	0.0314 ± 0.00855	<10^−3^	49,533	0.0300 ± 0.0133	0.0236
	Model 3	103,704	0.0289 ± 0.00748	<10^−3^	54,171	0.0304 ± 0.00862	<10^−3^	49,533	0.0256 ± 0.0133	0.0542

Quantile (median) regression models include different covariates. Model 1 (minimal) include age and sex only, and with dietary energy intake as a covariate for estimated food intake. Model 2 (primary model) includes age, sex, and dietary energy intake covariates from Model 1 and adds Townsend deprivation level of recruiting center, education, household income, smoking status, alcohol intake frequency, medication use (antihypertensive medications, cholesterol-lowering medications, aspirin, and insulin), and physical activity. Model 2 for HDL and triglycerides also includes fasting time before blood collection. Model 3 includes covariates in Model 2 plus 40 principal components (eigenvectors) to account for population stratification of genomic elements. Dietary manganese includes subjects with at least one in-person or online dietary entry for dietary manganese and energy intake. Results from Table 2 and Table 3 are from the same regression but display results for different predictors in the model. Correction for multiple comparisons performed with the Benjamini–Hochberg method.

**Table 3:** Association between dietary manganese with BMI, circulating HDL and triglycerides.

	Model	Both Sexes			Female Only			Male Only		
		N	Dietary Mn (Beta ± SE)	FDR Dietary Mn	N	Dietary Mn (Beta ± SE)	FDR Dietary Mn	N	Dietary Mn (Beta ± SE)	FDR Dietary Mn
BMI	Model 1	121,316	−0.566 ± 0.0112	<10^−300^	65,275	−0.613 ± 0.0182	<10^−200^	55,995	−0.525 ± 0.0140	<10^−300^
	Model 2	108,691	−0.531 ± 0.0118	<10^−300^	56,777	−0.597 ± 0.0190	<10^−200^	51,914	−0.458 ± 0.0140	<10^−200^
	Model 3	108,691	−0.524 ± 0.0116	<10^−300^	56,777	−0.595 ± 0.0192	<10^−200^	51,914	−0.471 ± 0.0142	<10^−200^
HDL (mmol/L)	Model 1	105,853	0.00887 ± 0.00106	<10^−15^	56,519	0.0137 ± 0.00176	<10^−10^	49,334	0.00652 ± 0.00124	<10^−5^
	Model 2	94,896	0.00958 ± 0.00103	<10^−15^	49,134	0.0138 ± 0.00167	<10^−15^	45,762	0.00763 ± 0.00121	<10^−5^
	Model 3	94,896	0.00973 ± 0.00104	<10^−15^	49,134	0.0146 ± 0.00166	<10^−15^	45,762	0.00744 ± 0.00123	<10^−5^
Triglycerides (mmol/L)	Model 1	115,768	−0.482 ± 0.00220	<10^−106^	62,282	−0.0396 ± 0.00262	<10^−50^	53,409	−0.0569 ± 0.00364	<10^−50^
	Model 2	103,763	−0.0451 ± 0.00229	<10^−50^	54,171	−0.0349 ± 0.00277	<10^−30^	49,533	−0.0517 ± 0.00375	<10^−30^
	Model 3	103,763	−0.0456 ± 0.00225	<10^−50^	54,171	−0.0344 ± 0.00279	<10^−30^	49,533	−0.0536 ± 0.00374	<10^−30^

Quantile (median) regression models include different covariates. Model 1 (minimal) include age and sex only, and with dietary energy intake as a covariate for estimated food intake. Model 2 (primary model) includes age, sex, and dietary energy intake covariates from Model 1 and adds Townsend deprivation level of recruiting center, education, household income, smoking status, alcohol intake frequency, medication use (antihypertensive medications, cholesterol-lowering medications, aspirin, and insulin), and physical activity. Model 2 for HDL and triglycerides also includes fasting time before blood collection. Model 3 includes covariates in Model 2 plus 40 principal components (eigenvectors) to account for population stratification of genomic elements. Dietary manganese includes subjects with at least one in-person or online dietary entry for dietary manganese and energy intake. Results from Table 2 and Table 3 are from the same regression but display results for different predictors in the model. Correction for multiple comparisons performed with the Benjamini–Hochberg method.

**Table 4:** Association of SLC39A8 SNP rs13107325 with measures of body composition, as measured by anthropometry and body impedance.

Anthropometric and Body Composition Measures	Both Sexes			Female Only			Male Only		
	N	rs13107325 (Beta ± SE)	*p*-Values/FDR rs13107325	N Female	rs13107325 (Beta ± SE)	*p*-Values/FDR rs13107325	N Male	rs13107325 (Beta ± SE)	*p*-Values/FDR rs13107325
Basal metabolic rate (kJ)	107,320	20.04 ± 7.36	0.00646	56,110	27.0 ± 8.24	0.00105	51,247	18.9 ± 13.4	0.159
Waist circumference (cm)	108,804	0.351 ± 0.106	<10^−3^	56,865	0.266 ± 0.161	0.0975	51,976	0.370 ± 0.139	0.00782
Body fat percent	107,247	0.39 ± 0.0611	<10^−5^	56,111	0.350 ± 0.0950	<10^−3^	51,173	0.418 ± 0.0765	<10^−5^
Whole body fat mass	107,123	0.467 ± 0.0771	<10^−5^	56,097	0.495 ± 0.121	<10^−3^	51,063	0.476 ± 0.100	<10^−5^
Whole body fat-free mass	107,314	0.139 ± 0.0587	0.0193	56,111	0.223 ± 0.0607	<10^−3^	51,240	0.106 ± 0.107	0.387
Trunk fat percentage	107,251	0.471 ± 0.0676	<10^−10^	56,076	0.443 ± 0.106	<10^−3^	51,212	0.460 ± 0.0875	<10^−5^
Trunk fat mass	107,246	0.258 ± 0.0465	<10^−5^	56,074	0.230 ± 0.067	<10^−3^	51,209	0.280 ± 0.0642	<10^−3^
Trunk fat-free mass	107,230	0.0545 ± 0.0305	0.0741	56,063	0.0830 ± 0.033	0.0119	51,204	0.00779 ± 0.0572	0.892
Arm fat percentage (left)	107,281	0.456 ± 0.0691	<10^−5^	56,089	0.609 ± 0.117	<10^−5^	51,229	0.375 ± 0.0717	<10^−5^
Arm fat percentage (right)	107,295	0.434 ± 0.0679	<10^−5^	56,097	0.560 ± 0.122	<10^−3^	51,235	0.326 ± 0.0618	<10^−5^
Arm fat mass (left)	107,268	0.0288 ± 0.00482	<10^−5^	56,082	0.0351 ± 0.00781	<10^−3^	51,223	0.0246 ± 0.00521	<10^−5^
Arm fat mass (right)	107,287	0.0244 ± 0.00444	<10^−5^	56,089	0.0308 ± 0.00741	<10^−3^	51,235	0.0210 ± 0.00470	<10^−3^
Arm fat-free mass (left)	107,268	0.0110 ± 0.00444	0.0148	56,085	0.0133 ± 0.00448	0.00339	51,220	0.00373 ± 0.00786	0.673
Arm fat-free mass (right)	107,285	0.0106 ± 0.00404	0.0108	56,091	0.0108 ± 0.00404	0.00790	51,231	0.00703 ± 0.00793	0.422
Leg fat percentage (left)	107,305	0.252 ± 0.0500	<10^−5^	56,104	0.227 ± 0.0730	0.00229	51,238	0.268 ± 0.0644	<10^−3^
Leg fat percentage (right)	107,316	0.238 ± 0.051	<10^−5^	56,107	0.272 ± 0.0748	<10^−3^	51,246	0.259 ± 0.0711	<10^−3^
Leg fat mass (left)	107,304	0.0541 ± 0.0117	<10^−5^	56,104	0.0618 ± 0.0189	0.00136	51,237	0.0512 ± 0.014	<10^−3^
Leg fat mass (right)	107,315	0.0589 ± 0.0119	<10^−5^	56,107	0.0688 ± 0.0194	<10^−3^	51,245	0.0490 ± 0.0143	<10^−3^
Leg fat-free mass (left)	107,299	0.0359 ± 0.011	0.00137	56,100	0.0402 ± 0.0115	<10^−3^	51,236	0.0295 ± 0.0184	0.152
Leg fat-free mass (right)	107,308	0.0470 ± 0.0107	<10^−3^	56,106	0.0521 ± 0.0114	<10^−3^	51,239	0.0270 ± 0.0194	0.211

Quantile (median) regression models (Model 2 used for all) include age, sex, Townsend deprivation level of recruiting center, education, household income, smoking status, alcohol intake frequency, medication use (antihypertensive medications, cholesterol-lowering medications, aspirin, and insulin), physical activity, and dietary energy intake. Dietary manganese includes subjects with at least one in-person or online dietary entry for dietary manganese and energy intake. Results from Table 4 and Table 5 are from the same regression but display results for different predictors in the model. All body composition measures by impedance are in kilograms. Correction for multiple comparisons performed with the Benjamini–Hochberg method for body composition measures by impedance.

**Table 5:** Associations of dietary manganese intake have opposing with measures of body composition, as measured by anthropometry and body impedance.

Anthropometric and Body Composition Measures	Both Sexes			Female Only			Male Only		
	N	Dietary Mn (Beta ± SE)	*p*-Values/FDR Dietary Mn	N Female	Dietary Mn (Beta ± SE)	*p*-Values/FDR Dietary Mn	N Male	Dietary Mn (Beta ± SE)	*p*-Values/FDR Dietary Mn
Basal metabolic rate (kJ)	107,320	−53.2 ± 2.22	<10^−100^	56,073	−50.05 ± 2.67	<10^−50^	51,247	−52.7 ± 3.78	<10^−30^
Waist circumference (cm)	108,804	−1.43 ± 0.0319	<10^−300^	56,828	−1.58 ± 0.0521	<10^−150^	51,976	−1.31 ± 0.0392	<10^−200^
Body fat percent	107,247	−0.839 ± 0.0184	<10^−300^	56,074	−0.950 ± 0.0307	<10^−200^	51,173	−0.764 ± 0.0216	<10^−250^
Whole body fat mass	107,123	−1.044 ± 0.0233	<10^−300^	56,060	−1.15 ± 0.0392	<10^−150^	51,063	−0.929 ± 0.0284	<10^−200^
Whole body fat-free mass	107,314	−0.314 ± 0.0177	<10^−50^	56,074	−0.297 ± 0.0197	<10^−50^	51,240	−0.311 ± 0.0302	<10^−20^
Trunk fat percentage	107,251	−0.897 ± 0.0204	<10^−300^	56,039	−0.969 ± 0.0344	<10^−150^	51,212	−0.838 ± 0.0247	<10^−200^
Trunk fat mass	107,246	−0.605 ± 0.014	<10^−300^	56,037	−0.610 ± 0.0217	<10^−150^	51,209	−0.594 ± 0.0181	<10^−200^
Trunk fat-free mass	107,230	−0.111 ± 0.0092	<10^−30^	56,026	−0.121 ± 0.0107	<10^−20^	51,204	−0.0936 ± 0.0161	<10^−5^
Arm fat percentage (left)	107,281	−0.821 ± 0.0208	<10^−300^	56,052	−1.21 ± 0.0379	<10^−200^	51,229	−0.639 ± 0.0202	<10^−200^
Arm fat percentage (right)	107,295	−0.750 ± 0.0205	<10^−250^	56,060	−1.21 ± 0.0397	<10^−200^	51,235	−0.554 ± 0.0174	<10^−200^
Arm fat mass (left)	107,268	−0.058 ± 0.00145	<10^−300^	56,045	−0.0772 ± 0.00253	<10^−200^	51,223	−0.0457 ± 0.00147	<10^−200^
Arm fat mass (right)	107,287	−0.0524 ± 0.00134	<10^−300^	56,052	−0.0711 ± 0.0024	<10^−150^	51,235	−0.0404 ± 0.00133	<10^−200^
Arm fat-free mass (left)	107,268	−0.0284 ± 0.00134	<10^−50^	56,048	−0.0263 ± 0.00145	<10^−50^	51,220	−0.0298 ± 0.00222	<10^−30^
Arm fat-free mass (right)	107,285	−0.0257 ± 0.00122	<10^−50^	56,054	−0.0197 ± 0.00131	<10^−50^	51,231	−0.0331 ± 0.00224	<10^−30^
Leg fat percentage (left)	107,305	−0.707 ± 0.0151	<10^−300^	56,067	−0.801 ± 0.0237	<10^−200^	51,238	−0.625 ± 0.0182	<10^−250^
Leg fat percentage (right)	107,316	−0.756 ± 0.01538	<10^−300^	56,070	−0.817 ± 0.0242	<10^−200^	51,246	−0.697 ± 0.0201	<10^−250^
Leg fat mass (left)	107,304	−0.152 ± 0.00353	<10^−300^	56,067	−0.191 ± 0.00612	<10^−200^	51,237	−0.124 ± 0.00396	<10^−200^
Leg fat mass (right)	107,315	−0.159 ± 0.00359	<10^−300^	56,070	−0.195 ± 0.0063	<10^−200^	51,245	−0.131 ± 0.00405	<10^−200^
Leg fat-free mass (left)	107,299	−0.0766 ± 0.00331	<10^−100^	56,063	−0.0614 ± 0.00372	<10^−50^	51,236	−0.0854 ± 0.00520	<10^−50^
Leg fat-free mass (right)	107,308	−0.0674 ± 0.00321	<10^−50^	56,069	−0.0582 ± 0.00371	<10^−50^	51,239	−0.0702 ± 0.00549	<10^−30^

Quantile (median) regression models (Model 2 used for all) include age, sex, Townsend deprivation level of recruiting center, education, household income, smoking status, alcohol intake frequency, medication use (antihypertensive medications, cholesterol-lowering medications, aspirin, and insulin), physical activity, and dietary energy intake. Dietary manganese includes subjects with at least one in-person or online dietary entry for dietary manganese and energy intake. All body composition measures by impedance are in kilograms. Results from Table 4 and Table 5 are from the same regression but display results for different predictors in the model. Correction for multiple comparisons performed with the Benjamini–Hochberg method for body composition measures by impedance.

**Table 6:** Association of SLC39A8 SNP rs13107325 with lipoproteins and lipid biomarkers.

Biomarker	Both Sexes			Female Only			Male Only		
	N	rs13107325 (Beta ± SE)	FDR rs13107325	N	rs13107325 (Beta ± SE)	FDR rs13107325	N	rs13107325 (Beta ± SE)	FDR rs13107325
HDL (mmol/L)	94,896	−0.0298 ± 0.00343	<10^−15^	49,134	−0.0382 ± 0.00515	<10^−10^	45,762	−0.0242 ± 0.00429	<10^−5^
ApoA (g/L)	94,335	−0.0170 ± 0.00241	<10^−10^	48,636	−0.0187 ± 0.00368	<10^−5^	45,699	−0.0169 ± 0.00323	<10^−5^
LDL (mmol/L)	103,571	−0.0191 ± 0.00811	0.0327	54,103	−0.0196 ± 0.0115	0.155	49,468	−0.0195 ± 0.0111	0.137
ApoB (g/L)	103,304	0.00106 ± 0.00227	0.745	54,023	−0.000158 ± 0.00303	0.959	49,281	−0.00283 ± 0.00323	0.444
Triglyceride (mmol/L)	103,704	0.0308 ± 0.00761	<10^−3^	54,171	0.0314 ± 0.00855	<10^−3^	49,533	0.0300 ± 0.0133	0.0550
Lipoprotein A (nmol/L)	82,815	−0.169 ± 0.335	0.745	43,397	0.437 ± 0.489	0.521	39,418	−0.678 ± 0.467	0.204
CRP (mg/L)	103,561	−0.00124 ± 0.0128	0.922	54,095	−0.0105 ± 0.0196	0.692	49,466	0.00700 ± 0.0168	0.676

Quantile (median) regression models (Model 2 used for all) include age, sex, Townsend deprivation level of recruiting center, education, household income, smoking status, alcohol intake frequency, medication use (antihypertensive medications, cholesterol-lowering medications, aspirin, and insulin), physical activity, dietary energy intake, and fasting time. HDL (Model 2) data is also listed in Table 2 and Table 3 and presented again here for reference. Dietary manganese includes subjects with at least one in-person or online dietary entry for dietary manganese and energy intake. Results from Table 6 and Table 7 are from the same regression but display results for different predictors in the model. Correction for multiple comparisons performed with the Benjamini–Hochberg method.

**Table 7:** Association of dietary manganese intake with lipoproteins and lipid biomarkers.

Biomarker	Both Sexes			Female Only			Male Only		
	N	Dietary Mn (Beta ± SE)	FDR Dietary Mn	N	Dietary Mn (Beta ± SE)	FDR Dietary Mn	N	Dietary Mn (Beta ± SE)	FDR Dietary Mn
HDL (mmol/L)	94,896	0.00958 ± 0.00103	<10^−15^	49,134	0.0139 ± 0.00167	<10^−15^	45,762	0.00763 ± 0.00121	<10^−5^
ApoA (g/L)	94,335	0.00128 ± 0.000726	0.778	48,636	0.00159 ± 0.00119	0.183	45,699	0.00117 ± 0.000913	0.199
LDL (mmol/L)	103,571	−0.0520 ± 0.00245	<10^−50^	54,103	−0.0476 ± 0.00373	<10^−30^	49,468	−0.0472 ± 0.00313	<10^−50^
ApoB (g/L)	103,304	−0.0157 ± 0.000684	<10^−100^	54,023	−0.0151 ± 0.000983	<10^−50^	49,281	−0.0144 ± 0.000911	<10^−50^
Triglyceride (mmol/L)	103,704	−0.0451 ± 0.00229	<10^−50^	54,171	−0.0349 ± 0.00277	<10^−30^	49,533	−0.0517 ± 0.00375	<10^−30^
Lipoprotein A (nmol/L)	82,815	0.430 ± 0.101	<10^−3^	43,397	0.519 ± 0.159	0.00125	39,418	0.404 ± 0.131	0.00245
CRP (mg/L)	103,561	−0.126 ± 0.00385	<10^−200^	54,095	−0.141 ± 0.00635	<10^−100^	49,466	−0.112 ± 0.00473	<10^−100^

Quantile (median) regression models (Model 2 used for all) include age, sex, Townsend deprivation level of recruiting center, education, household income, smoking status, alcohol intake frequency, medication use (antihypertensive medications, cholesterol-lowering medications, aspirin, and insulin), physical activity, dietary energy intake, and fasting time. HDL (Model 2) data is also listed in Table 1 and presented again here for reference. Dietary manganese includes subjects with at least one in-person or online dietary entry for dietary manganese and energy intake. Results from Table 6 and Table 7 are from the same regression but display results for different predictors in the model. Correction for multiple comparisons performed with the Benjamini–Hochberg method.

**Table 8:** Association of SLC39A8 SNP rs13107325 and dietary manganese intake with lipoprotein particle properties and triglyceride/HDL subfraction composition as determined by NMR metabolomics.

Lipid/Lipoprotein Composition	Both Sexes			
	rs13107325 (Beta ± SE)	FDR rs13107325	Dietary Mn (Beta ± SE)	FDR Dietary Mn
Average Diameter for HDL Particles	−0.0108 ± 0.00240	<10^−3^	0.0129 ± 0.000724	<10^−50^
Average Diameter for LDL Particles	−0.00316 ± 0.00115	0.00911	0.00351 ± 0.000346	<10^−20^
Average Diameter for VLDL Particles	0.0575 ± 0.0168	0.00119	−0.0703 ± 0.005071	<10^−30^
Concentration of HDL Particles	−0.0000938 ± 0.0000283	0.00162	−0.0000462 ± 0.000008536	<10^−5^
Concentration of Large HDL Particles	−0.0000392 ± 0.00000857	<10^−3^	0.0000395 ± 0.000002587	<10^−50^
Concentration of Medium HDL Particles	−0.0000372 ± 0.0000106	0.00105	−0.00000854 ± 0.000003209	0.00929
Concentration of Small HDL Particles	−0.0000193 ± 0.0000165	0.276	−0.000092 ± 0.000004992	<10^−50^
Concentration of Very Large HDL Particles	−0.00000492 ± 0.00000101	<10^−5^	0.00000439 ± 0.000000305	<10^−30^
Triglycerides in Chylomicrons and Extremely Large VLDL	0.00428 ± 0.00152	0.00761	−0.006562 ± 0.000457	<10^−30^
Triglycerides in HDL	0.000508 ± 0.000574	0.400	−0.002724 ± 0.000173	<10^−50^
Triglycerides in IDL	0.000576 ± 0.00031	0.0854	−0.00155 ± 0.0000935	<10^−50^
Triglycerides in LDL	0.000846 ± 0.00046	0.0859	−0.00267 ± 0.000139	<10^−50^
Triglycerides in Large HDL	−0.000125 ± 0.000152	0.424	−0.000236 ± 0.0000458	<10^−5^
Triglycerides in Medium HDL	0.000389 ± 0.000233	0.116	−0.00117 ± 0.0000704	<10^−50^
Triglycerides in Small HDL	0.000759 ± 0.000214	<10^−3^	−0.00135 ± 0.0000648	<10^−50^
Triglycerides in VLDL	0.0124 ± 0.00613	0.0632	−0.0292 ± 0.001852	<10^−50^
Triglycerides in Very Large HDL	−0.0000326 ± 0.00003	0.312	−0.000054 ± 0.0000092	<10^−5^
Triglycerides to Total Lipids in Large HDL percentage	0.122 ± 0.0326	<10^−3^	−0.166 ± 0.009831	<10^−50^
Triglycerides to Total Lipids in Medium HDL percentage	0.0769 ± 0.0223	0.00119	−0.108 ± 0.006725	<10^−50^
Triglycerides to Total Lipids in Small HDL percentage	0.0603 ± 0.0158	<10^−3^	−0.0668 ± 0.004774	<10^−30^
Triglycerides to Total Lipids in Very Large HDL percentage	0.137 ± 0.0307	<10^−3^	−0.178 ± 0.009278	<10^−50^
Cholesterol in Large HDL	−0.009701 ± 0.00181	<10^−5^	0.00935 ± 0.000546	<10^−50^
Cholesterol in Medium HDL	−0.00485 ± 0.00142	0.00119	0.000351 ± 0.000428	0.419
Cholesterol in Small HDL	−0.000969 ± 0.000773	0.247	−0.00391 ± 0.000233	<10^−50^
Cholesterol in Very Large HDL	−0.00174 ± 0.000368	<10^−3^	0.00188 ± 0.000111	<10^−50^
Cholesterol to Total Lipids in Large HDL percentage	−0.326 ± 0.0574	<10^−5^	0.396 ± 0.0173	<10^−100^
Cholesterol to Total Lipids in Medium HDL percentage	−0.139 ± 0.0387	<10^−3^	0.178 ± 0.0117	<10^−50^
Cholesterol to Total Lipids Small HDL percentage	−0.0420 ± 0.0238	0.0972	0.074 ± 0.0072	<10^−20^
Cholesterol to Total Lipids Very Large HDL percentage	0.0956 ± 0.0488	0.0707	−0.167 ± 0.0147	<10^−20^
Total Lipids in HDL	−0.0349 ± 0.00711	<10^−5^	0.00532 ± 0.00215	0.123
Total Lipids in Large HDL	−0.0163 ± 0.00360	<10^−3^	0.0154 ± 0.00109	<10^−30^
Total Lipids in Medium HDL	−0.00800 ± 0.0026	0.00364	−0.0031 ± 0.000786	<10^−3^
Total Lipids in Small HDL	0.000360 ± 0.00199	0.856	−0.0124 ± 0.0006	<10^−50^
Total Lipids in Very Large HDL	−0.00413 ± 0.000867	<10^−3^	0.00426 ± 0.000262	<10^−50^

Quantile (median) regression models (Model 2 used for all) include age, sex, Townsend deprivation level of recruiting center, education, household income, smoking status, alcohol intake frequency, medication use (antihypertensive medications, cholesterol-lowering medications, aspirin, and insulin), physical activity, dietary energy intake, and fasting time. Dietary manganese includes subjects with at least one in-person or online dietary entry for dietary manganese and energy intake. N = 59,663 for both sexes, except for triglycerides to total lipids in very large HDL percentage (N = 59,634). Correction for multiple comparisons performed with the Benjamini–Hochberg method.

**Table 9:** Association of SLC39A8 SNP rs13107325 with blood pressure.

BP (mm Hg)	Both Sexes			Female Only			Male Only		
	N	rs13107325 (Beta ± SE)	*p*-Values rs13107325	N	rs13107325 (Beta ± SE)	*p*-Values rs13107325	N	rs13107325 (Beta ± SE)	*p*-Values rs13107325
Systolic BP	104,684	−0.601 ± 0.172	<10^−3^	54,646	−0.711 ± 0.246	0.00386	50,038	−0.413 ± 0.235	0.0791
Diastolic BP	104,684	−0.531 ± 0.100	<10^−5^	54,646	−0.514 ± 0.139	<10^−3^	50,038	−0.528 ± 0.148	<10^−3^

Quantile regression models (Model 2 used for all) include age, sex, Townsend deprivation level of recruiting center, education, household income, smoking status, alcohol intake frequency, medication use (antihypertensive medications, cholesterol-lowering medications, aspirin, and insulin), physical activity, dietary energy intake, and fasting time. Results from Table 9 and Table 10 are from the same regression but display results for different predictors in the model. Dietary manganese includes subjects with at least one in-person or online dietary entry for dietary manganese and energy intake.

**Table 10:** Association of dietary manganese intake with blood pressure.

BP (mm Hg)	Both Sexes			Female Only			Male Only		
	N	Dietary Mn (Beta ± SE)	*p*-Values Dietary Mn	N	Dietary Mn (Beta ± SE)	*p*-Values Dietary Mn	N	Dietary Mn (Beta ± SE)	*p*-Values Dietary Mn
Systolic BP	104,684	−0.529 ± 0.0520	<10^−20^	54,646	−0.549 ± 0.0800	<10^−10^	50,038	−0.485 ± 0.0664	<10^−10^
Diastolic BP	104,684	−0.562 ± 0.0302	<10^−50^	54,646	−0.584 ± 0.0452	<10^−30^	50,038	−0.521 ± 0.0418	<10^−30^

Quantile regression models (Model 2 used for all) include age, sex, Townsend deprivation level of recruiting center, education, household income, smoking status, alcohol intake frequency, medication use (antihypertensive medications, cholesterol-lowering medications, aspirin, and insulin), physical activity, dietary energy intake, and fasting time. Results from Table 9 and Table 10 are from the same regression but display results for different predictors in the model. Dietary manganese includes subjects with at least one in-person or online dietary entry for dietary manganese and energy intake.

## Data Availability

The data presented in this study was accessed on 17 January 2024 from UK Biobank at https://www.ukbiobank.ac.uk. The UK Biobank data can be accessed by researchers with approved application.

## Supplementary Materials

The following supporting information can be downloaded at: https://www.mdpi.com/article/10.3390/nu17193031/s1, Figure S1: Flowchart of exclusion criteria used to produce final UK Biobank cohort for analyses. For genetic association studies with *SLC39A8* SNP rs13107325, the final cohort was limited to participants classified as Caucasian (variable ID 22006) and without kinship (variable ID 22021) by excluding individuals outside of Caucasian genetic grouping or with genetic relatives within the cohort, respectively. These filters reduced the final cohort from 502,233 to 276,436 subjects; Table S1: List of variables and variable identification numbers used; Table S2: Dietary manganese intake is associated with lower BMI and circulating triglycerides and improvements in HDL in UK Biobank participants with all four online dietary recalls; Table S3: Opposing associations between *SLC39A8* SNP rs13107325 and dietary manganese intake remain in participants reporting typical diets for recorded dietary recalls; Table S4: Opposing associations between *SLC39A8* SNP rs13107325 and dietary manganese intake remain in participants not reporting vitamin/mineral supplement use for recorded dietary recalls; Table S5: Opposing associations between *SLC39A8* SNP rs13107325 and dietary manganese intake remain after inclusion of dietary covariates often associated with dietary manganese; Table S6: Consideration of interactions between rs13107325 and dietary manganese in associations with BMI and circulating HDL and triglycerides; Table S7: Dietary manganese intake is associated with reduced adiposity in measures of body composition in UK Biobank participants with all four online dietary recalls; Table S8: Dietary manganese intake is associated with reduced adiposity as measured with abdominal imaging using dual energy X-ray absorptiometry (DEXA) or magnetic resonance imaging (MRI) in UK Biobank participants; Table S9: Dietary manganese intake is associated with lipoprotein particle size, concentrations, and composition, as determined by NMR metabolomics for males and females separately; Table S10: Dietary manganese intake is associated with lipoprotein particle size, concentrations, and composition, as determined by NMR metabolomics, in UK Biobank participants with all four online dietary recalls.

## Abbreviations

The following abbreviations are used in this manuscript:

AI	Adequate Intake
ApoA	Apolipoprotein A
ApoB	Apolipoprotein B
BMI	Body Mass Index
CRP	*C*-reactive Protein
CVD	Cardiovascular Disease
DEXA	Dual X-ray Absorptiometry
FDR	False Discovery Rate
GWAS	Genome-wide Association Studies
HDL	High Density Lipoprotein
LDL	Low Density Lipoprotein
MRI	Magnetic Resonance Imaging
NMR	Nuclear Magnetic Resonance
NT-proBNP	*N*-terminal Prohormone of Brain Natriuretic Peptide
RAP	Research Access Platform
SNP	Single Nucleotide Polymorphism
VWF	von Willebrand factor
