# Supplementary material for: Metal Transporter Gene SLC39A8 Polymorphism rs13107325 and Dietary Manganese Intake Are Associated with Measures of Cardiovascular Disease Risk in a UK Biobank Population Cohort
Source: Nutrients. 2025 Sep 23;17(19):3031. doi: 10.3390/nu17193031 (PMC12525855; doi:10.3390/nu17193031)
Supplement: Supplementary file 1 [file nutrients-17-03031-s001.zip › nutrients-3844072-supplementary.pdf]

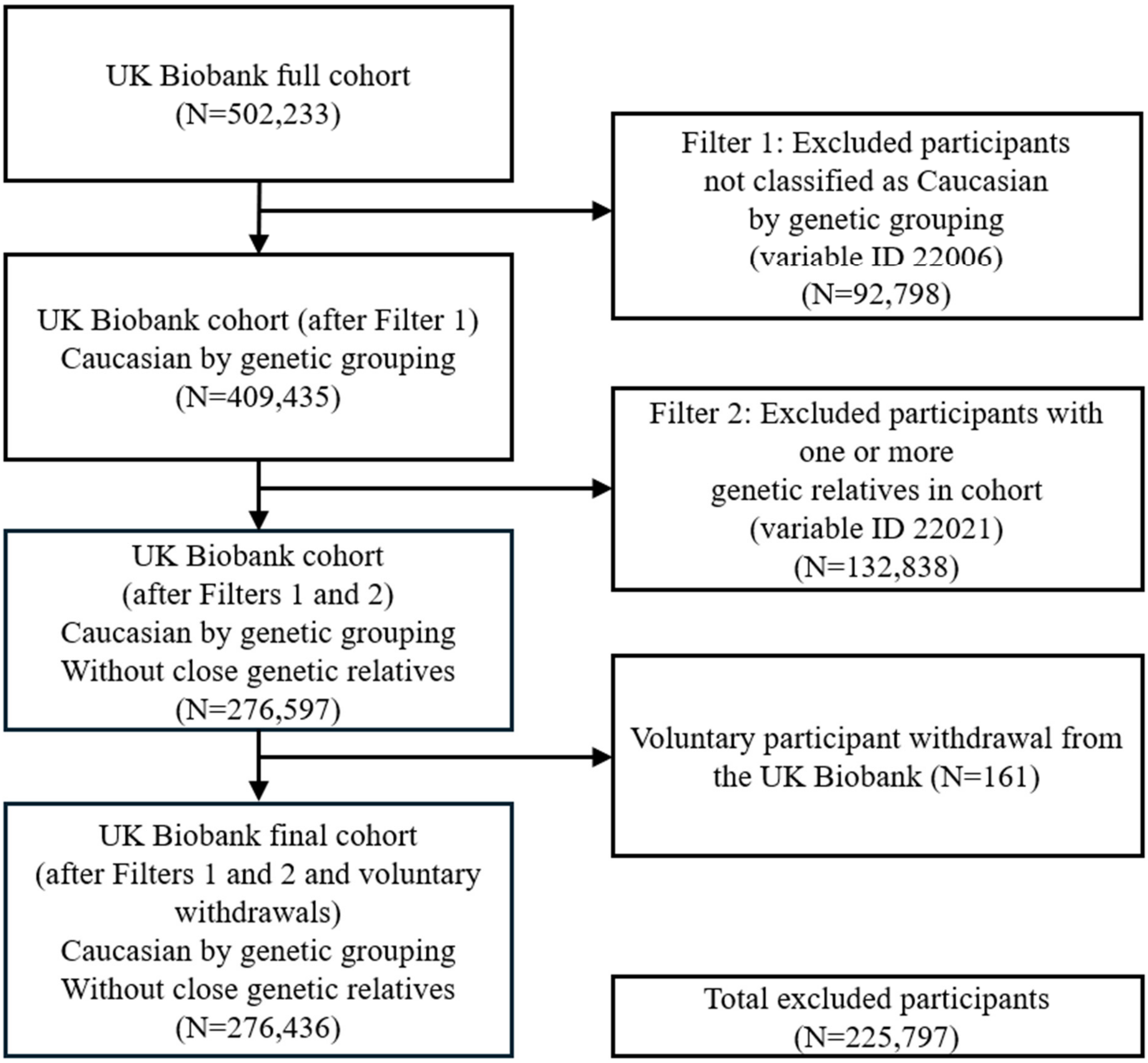

Supplemental Figure S1. Flowchart of exclusion criteria used to produce final UK Biobank cohort for analyses. For genetic association studies with *SLC39A8* SNP rs13107325, the final cohort was limited to participants classified as Caucasian (variable ID 22006) and without kinship (variable ID 22021) by excluding individuals outside of Caucasian genetic grouping or with genetic relatives within the cohort, respectively. These filters reduced the final cohort from 502,233 to 276,436 subjects.

Supplemental Table S1. List of variables and variable identification numbers used

**Variable term**	**UK Biobank Variable ID**
Dietary manganese (24-hour) intake	26051
Energy intake	26002
Elemental iron (non-heme)	26053
Heme iron	26046
Zinc	26033
Selenium	26058
Englyst dietary fiber	26017
First in-person dietary record	instance 0
Four subsequent online surveys	instances 1-4
Daily dietary data not credible	100026
Dietary intake not typical	100020
Vitamin or mineral supplement use	104670
Body mass index (BMI)	21001
Body fat percentage	23099
High density lipoprotein (HDL)	30760
Low density lipoprotein (LDL)	30780
Triglycerides	30870
Fasting time (before blood collection)	74
Blood pressure: systolic	4080
Blood pressure: diastolic	4079
Body impedance	23111-23130
Plasma nuclear magnetic resonance (NMR) metabolomics	23407- 23648
Dual x-ray absorptiometry (DEXA)	21110 - 23289
Magnetic resonance imaging (MRI)	22407 - 22435
Education level (qualifications)	6138
Household income before tax	738
Townsend deprivation index	22189
Age (at enrollment)	21022
Sex	31
Tobacco smoking status (at enrollment)	20116
Summed Metabolic Equivalent Task (MET) minutes per week for all activity	22040
Alcohol intake frequency (at enrollment)	1558
Medication use (at enrollment)	6153, 6177, 6154
Genetic principal components (40 provided eigenvectors)	22009
Caucasian genetic ancestral grouping	22006
Genetic kinship to other participants	22021

Instances indicate different timepoints or occurrences at which data was collected within that variable.

Supplemental Table S2. Dietary manganese intake is associated with lower BMI and circulating triglycerides and improvement in HDL in UK Biobank participants with all four online dietary recalls.

	**N**	**rs13107325 (beta ± SE)**	**FDR rs13107325**	**Dietary Mn (beta ± SE)**	**FDR Dietary Mn**
**BMI**					
Model 1	16,032	0.219±0.103	0.492	-0.744±0.0341	$<10^{-100}$
Model 2	14,608	0.180±0.105	0.131	-0.674±0.0354	$<10^{-50}$
Model 3	14,608	0.203±0.105	0.0801	-0.676±0.0354	$<10^{-50}$
**HDL (mmol/L)**					
Model 1	13,989	-0.0256±0.0101	0.0325	0.0154±0.00332	$<10^{-05}$
Model 2	12,770	-0.0239±0.0101	0.0533	0.0167±0.00339	$<10^{-05}$
Model 3	12,770	-0.0262±0.00101	0.0291	0.0157±0.00400	$<10^{-05}$
**Triglycerides (mmol/L)**					
Model 1	15,238	0.00971±0.0202	0.631	-0.0622±0.00670	$<10^{-15}$
Model 2	13,895	0.0175±0.0208	0.400	-0.0640±0.00699	$<10^{-15}$
Model 3	13,895	0.0207±0.0209	0.323	-0.0650±0.00703	$<10^{-15}$

Quantile (median) regression models include different covariates. Model 1 (minimal) include age and sex. Model 2 (primary model) includes age, sex, Townsend deprivation level of recruiting center, education, household income, smoking status, alcohol intake frequency, medication use (antihypertensive medications, cholesterol-lowering medications, aspirin, and insulin), and physical activity. Model 2 for HDL and triglyceride also includes fasting time before blood collection. Both sexes are used in analyses. Correction for multiple comparisons performed with the Benjamini-Hochberg method.

Supplemental Table S3. Opposing associations between *SLC39A8* SNP rs13107325 and dietary manganese intake remain in participants reporting typical diets for recorded dietary recalls.

**Health Measure**	**N**	**rs13107325 (beta ± SE)**	**FDR rs13107325**	**Dietary Mn (beta ± SE)**	**FDR Dietary Mn**
BMI	99,479	0.289±0.0408	$<10^{-10}$	-0.490± 0.0120	$<10^{-300}$
HDL	86,875	-0.0298±0.00361	$<10^{-15}$	0.00873±0.00106	$<10^{-15}$
Triglyceride	94,932	0.0336±0.00798	$<10^{-03}$	-0.0433±0.00235	$<10^{-50}$

Quantile (median) regression model (Model 2) includes age, sex, Townsend deprivation level of recruiting center, education, household income, smoking status, alcohol intake frequency, medication use (antihypertensive medications, cholesterol-lowering medications, aspirin, and insulin), and physical activity. Model 2 for HDL and triglyceride also includes fasting time before blood collection. Only dietary records self-reported as typical diets were included in the analyses. Subjects of both sexes include participants with at least one in-person or online dietary recall. Correction for multiple comparisons performed with the Benjamini-Hochberg method.

Supplemental Table S4. Opposing associations between *SLC39A8* SNP rs13107325 and dietary manganese intake remain in participants not reporting vitamin/mineral supplement use for recorded dietary recalls.

**Health Measure**	**N**	**rs13107325 (beta ± SE)**	**FDR rs13107325**	**Dietary Mn (beta ± SE)**	**FDR Dietary Mn**
BMI	71,038	0.241±0.0497	$<10^{-05}$	-0.507± 0.0148	$<10^{-200}$
HDL	61,999	-0.0271±0.00420	$<10^{-05}$	0.0101±0.00125	$<10^{-15}$
Triglyceride	67,788	0.0365±0.00974	$<10^{-03}$	-0.0445±0.00289	$<10^{-50}$

Quantile (median) regression model (Model 2) includes age, sex, Townsend deprivation level of recruiting center, education, household income, smoking status, alcohol intake frequency, medication use (antihypertensive medications, cholesterol-lowering medications, aspirin, and insulin), and physical activity. Model 2 for HDL and triglyceride also includes fasting time before blood collection. Dietary records with multivitamin-multimineral supplement use were excluded. Subjects of both sexes include participants with at least one in-person or online dietary recall. Correction for multiple comparisons performed with the Benjamini-Hochberg method.

Supplemental Table S5. Opposing associations between *SLC39A8* SNP rs13107325 and dietary manganese intake remain after inclusion of dietary covariates often associated with dietary manganese.

**Health Measure**	**Model**	**N**	**rs13107325 (beta ± SE)**	**FDR rs13107325**	**Dietary Mn (beta ± SE)**	**FDR Dietary Mn**
**BMI**	Model 2	108,691	0.283±0.0392	$<10^{-10}$	-0.531±0.0118	$<10^{-300}$
	Additional covariates	108,691	0.299±0.0391	$<10^{-10}$	-0.605±0.0178	$<10^{-200}$
**HDL**	Model 2	94,896	-0.0300±0.00342	$<10^{-15}$	0.00958±0.00103	$<10^{-15}$
	Additional covariates	94,896	-0.0298±0.00343	$<10^{-15}$	0.0173±0.00156	$<10^{-20}$
**Triglyceride**	Model 2	103,763	0.0308±0.00760	$<10^{-03}$	-0.0451±0.00229	$<10^{-50}$
	Additional covariates	103,763	0.0333±0.00775	$<10^{-03}$	-0.0486±0.00353	$<10^{-30}$

Quantile (median) regression models (Model 2) include age, sex, Townsend deprivation level of recruiting center, education, household income, smoking status, alcohol intake frequency, medication use (antihypertensive medications, cholesterol-lowering medications, aspirin, and insulin), and physical activity. Model 2 for HDL and triglyceride also includes fasting time before blood collection. Additional dietary covariates added to Model 2 (if indicated) include zinc, bioavailable iron, selenium, and fiber. Subjects of both sexes include participants with at least one in-person or online dietary recall. Correction for multiple comparisons performed with the Benjamini-Hochberg method.

Supplemental Table S6. Consideration of interactions between rs13107325 and dietary manganese in associations with BMI and circulating HDL and triglycerides.

**Both Sexes**							
	**N both sexes**	**Interaction (beta ± SE)**	**Interaction p-values**	**rs13107325 (beta ± SE)**	**rs13107325 p-values**	**Dietary Mn (beta ± SE)**	**Dietary Mn p-values**
**BMI**	108,691	-0.00242±0.0266	0.927	0.294±0.119	0.0135	-0.530±0.0124	$<10^{-300}$
**HDL (mmol/L)**	94,896	0.00402±0.00233	0.0849	-0.0470±0.0104	$<10^{-05}$	0.00890±0.00109	$<10^{-15}$
**Triglycerides (mmol/L)**	103,704	-0.00970±0.00512	0.0585	0.0708±0.0229	0.00198	-0.0439±0.00239	$<1E^{-50}$
**Female only**							
	**N female**	**Interaction (beta ± SE)**	**Interaction p-values**	**rs13107325 (beta ± SE)**	**rs13107325 p-values**	**Dietary Mn (beta ± SE)**	**Dietary Mn p-values**
**BMI**	56,777	-0.0252±0.0423	0.552	0.344±0.182	0.0588	-0.593±0.0200	$<10^{-100}$
**HDL (mmol/L)**	49,134	0.00519±0.00383	0.176	-0.0612±0.0165	$<10^{-03}$	0.0165±0.00180	$<10^{-15}$
**Triglycerides (mmol/L)**	54,171	-0.0120±0.00646	0.0625	0.0801±0.0278	0.00397	-0.0229±0.00304	$<10^{-10}$
**Male only**							
	**N male**	**Interaction (beta ± SE)**	**Interaction p-values**	**rs13107325 (beta ± SE)**	**rs13107325 p-values**	**Dietary Mn (beta ± SE)**	**Dietary Mn p-values**
**BMI**	51,914	0.0195±0.0323	0.547	0.212±0.150	0.157	-0.473±0.0149	$<10^{-200}$
**HDL (mmol/L)**	45,762	0.00440±0.00277	0.113	-0.427±0.0129	$<10^{-03}$	0.00690±0.00127	$<10^{-05}$
**Triglycerides (mmol/L)**	49,533	0.000636±0.00859	0.941	0.0267±0.0398	0.502	-0.0519±0.00394	$<10^{-30}$

Quantile (median) regression models (Model 2 used for all) include age, sex, Townsend deprivation level of recruiting center, education, household income, smoking status, alcohol intake frequency, medication use (antihypertensive medications, cholesterol-lowering medications, aspirin, and insulin), and physical activity. Dietary manganese includes subjects with at least one in-person or online dietary entry for dietary manganese and energy intake.

Supplemental Table S7. Dietary manganese intake is associated with reduced adiposity in measures of body composition in UK Biobank participants with all four online dietary recalls.

**Anthropometric and body composition measures**	**Both sexes**					**Female only**					**Male only**				
	**N**	**rs13107325 (beta ± SE)**	**FDR rs13107325**	**Dietary Mn (beta ± SE)**	**FDR dietary Mn**	**N Female**	**rs13107325 (beta ± SE)**	**FDR rs13107325**	**Dietary Mn (beta ± SE)**	**FDR dietary Mn**	**N male**	**rs13107325 (beta ± SE)**	**FDR rs13107325**	**Dietary Mn (beta ± SE)**	**FDR dietary Mn**
Basal metabolic rate (kJ)	14,442	18.9±20.8	0.364	-67.8±7.0180	$<10^{-50}$	7,734	4.68±22.2	0.833	-65.1±8.06	$<10^{-15}$	6,708	45.9±39.4	0.348	-75.0±12.4	$<10^{-05}$
Waist circumference (cm)	14,612	0.0775±0.289	0.788	-1.83±0.0973	$<10^{-50}$	7,816	0.209±0.419	0.618	-1.95±0.152	$<10^{-30}$	6,796	0.0218±0.370	0.348	-1.69±0.116	$<10^{-30}$
Body fat percent	14,425	0.203±0.168	0.559	-1.13±0.0567	$<10^{-50}$	7,734	0.0443±0.256	0.910	-1.24±0.0930	$<10^{-30}$	6,691	0.349±0.216	0.348	-1.03±0.0680	$<10^{-30}$
Whole body fat mass	14,402	0.223±0.216	0.559	-1.35±0.0730	$<10^{-50}$	7,733	0.125±0.323	0.910	-1.42±0.118	$<10^{-30}$	6,669	0.397±0.293	0.348	-1.30±0.0924	$<10^{-30}$
Whole body fat-free mass	14,440	0.136±0.159	0.590	-0.416±0.0538	$<10^{-10}$	7,734	0.078±0.168	0.910	-0.384±0.061	$<10^{-05}$	6,706	0.239±0.304	0.486	-0.457±0.0958	$<10^{-05}$
Trunk fat percentage	14,432	0.191±0.185	0.559	-1.23±0.0625	$<10^{-50}$	7,730	-0.102±0.283	0.910	-1.32±0.103	$<10^{-30}$	6,702	0.363±0.246	0.348	-1.16±0.0773	$<10^{-30}$
Trunk fat mass	14,430	0.115±0.133	0.590	-0.822±0.0448	$<10^{-50}$	7,729	-0.0280±0.185	0.910	-0.797±0.0672	$<10^{-30}$	6,701	0.245±0.183	0.348	-0.866±0.0576	$<10^{-30}$
Trunk fat-free mass	14,424	0.0521±0.0858	0.652	-0.16±0.0289	$<10^{-05}$	7,725	0.0434±0.0880	0.910	-0.159±0.032	$<10^{-05}$	6,699	0.143±0.163	0.486	-0.153±0.0514	0.00296
Arm fat percentage (left)	14,438	0.216±0.192	0.559	-1.145±0.0647	$<10^{-50}$	7,730	0.119±0.314	0.910	-1.55±0.114	$<10^{-30}$	6,708	0.236±0.204	0.392	-0.894±0.0641	$<10^{-30}$
Arm fat percentage (right)	14,439	0.256±0.188	0.559	-1.039±0.0634	$<10^{-50}$	7,731	0.0547±0.319	0.910	-1.62±0.116	$<10^{-30}$	6,708	0.242±0.186	0.348	-0.736±0.0586	$<10^{-30}$
Arm fat mass (left)	14,436	0.0239±0.0128	0.559	-0.0754±0.00433	$<10^{-50}$	7,730	0.0212±0.0203	0.910	-0.0943±0.00740	$<10^{-30}$	6,706	0.0220±0.0159	0.348	-0.0605±0.005	$<10^{-30}$
Arm fat mass (right)	14,437	0.0150±0.0115	0.559	-0.0644±0.0039	$<10^{-50}$	7,729	0.0142±0.0183	0.910	-0.0853±0.00666	$<10^{-30}$	6,708	0.0110±0.0139	0.486	-0.0538±0.0044	$<10^{-30}$
Arm fat-free mass (left)	14,435	-0.00145±0.0120	0.904	-0.0421±0.00405	$<10^{-20}$	7,730	0.00367±0.0124	0.910	-0.0341±0.00451	$<10^{-10}$	6,705	-0.00338±0.0223	0.880	-0.0498±0.007	$<10^{-10}$
Arm fat-free mass (right)	14,438	0.00548±0.0110	0.695	-0.0361±0.00371	$<10^{-20}$	7,730	0.00494±0.0107	0.910	-0.0266±0.00390	$<10^{-10}$	6,708	0.0105±0.0204	0.644	-0.0478±0.00642	$<10^{-10}$
Leg fat percentage (left)	14,440	0.105±0.131	0.590	-0.976±0.0443	$<10^{-100}$	7,731	-0.182±0.190	0.910	-1.03±0.0693	$<10^{-30}$	6,709	0.282±0.182	0.348	-0.838±0.0571	$<10^{-30}$
Leg fat percentage (right)	14,442	0.179±0.143	0.559	-1.01±0.0483	$<10^{-50}$	7,733	-0.0667±0.198	0.910	-1.05±0.0720	$<10^{-30}$	6,709	0.345±0.194	0.348	-0.898±0.0611	$<10^{-30}$
Leg fat mass (left)	14,439	0.0322±0.0317	0.559	-0.198±0.0107	$<10^{-50}$	7,731	-0.00923±0.0494	0.910	-0.241±0.018	$<10^{-30}$	6,708	0.0493±0.0379	0.348	-0.172±0.0119	$<10^{-30}$
Leg fat mass (right)	14,442	0.0226±0.0333	0.640	-0.200±0.0112	$<10^{-50}$	7,733	0.0103±0.0515	0.910	-0.241±0.0187	$<10^{-30}$	6,709	0.0577±0.0408	0.348	-0.174±0.0128	$<10^{-30}$
Leg fat-free mass (left)	14,439	0.0109±0.0281	0.740	-0.092±0.00948	$<10^{-20}$	7,731	0.00350±0.0310	0.910	-0.0762±0.0113	$<10^{-10}$	6,708	0.0591±0.0526	0.392	-0.111±0.0165	$<10^{-10}$
Leg fat-free mass (right)	14,442	0.0394±0.0288	0.559	-0.0804±0.00970	$<10^{-15}$	7,733	0.0300±0.0314	0.910	-0.0746±0.0114	$<10^{-10}$	6,709	0.0511±0.0524	0.456	-0.0917±0.0165	$<10^{-05}$

Quantile (median) regression model (Model 2) includes age, sex, Townsend deprivation level of recruiting center, education, household income, smoking status, alcohol intake frequency, medication use (antihypertensive medications, cholesterol-lowering medications, aspirin, and insulin), and physical activity. Subjects include participants with entries for all four online dietary recalls.

Tissue masses are provided in kg. All body composition measures by impedance are in kilograms. Correction for multiple comparisons performed with the Benjamini-Hochberg method for body composition measures by impedance.

Supplemental Table S8. Dietary manganese intake is associated with reduced adiposity as measured with abdominal imaging using dual energy X-ray absorptiometry (DEXA) or magnetic resonance imaging (MRI) in UK Biobank participants.

**Body composition measures**	**Both sexes**					**Female only**					**Male only**				
	**N**	**rs13107325 (beta ± SE)**	**FDR rs13107325**	**Dietary Mn (beta ± SE)**	**FDR dietary Mn**	**N Female**	**rs13107325 (beta ± SE)**	**p-values rs13107325**	**Dietary Mn (beta ± SE)**	**FDR dietary Mn**	**N male**	**rs13107325 (beta ± SE)**	**p-values rs13107325**	**Dietary Mn (beta ± SE)**	**FDR dietary Mn**
**DEXA**															
Total fat mass	13,832	455±232	0.160	-1410±68.9	$<10^{-50}$	6,863	535±363	0.359	-1523±116	$<10^{-30}$	6,969	326±304	0.380	-1351±85.4	$<10^{-50}$
Total fat-free mass	13,832	95.7±160	0.653	-169±47.46	$<10^{-03}$	6,863	170±202	0.455	-243±64.6	$<10^{-03}$	6,969	71±252	0.778	-152±70.8	0.0416
Total lean mass	13,832	148±155	0.425	-150±46.16	0.00140	6,863	240±187	0.359	-202±60.0	$<10^{-03}$	6,969	78.6±248	0.778	-143±69.6	0.0498
Total tissue fat percentage	16,318	0.00382±0.00175	0.137	-0.0128±0.000518	$<10^{-100}$	8,113	0.00488±0.00262	0.260	-0.0137±0.000839	$<10^{-50}$	8,205	0.00429±0.00231	0.380	-0.0119±0.000642	$<10^{-50}$
Trunk fat mass	13,832	359±165	0.137	-1000±49.1	$<10^{-50}$	6,863	486±241	0.218	-1016±77.2	$<10^{-30}$	6,969	280±224	0.380	-967±62.7	$<10^{-50}$
Trunk lean mass	13,832	94.7±70.9	0.288	4.85±21.1	0.818	6,863	66.6±84.8	0.470	-18.3±27.1	0.499	6,969	154±114	0.380	34±32	0.336
Trunk tissue fat percentage	18,494	0.35±0.1639	0.137	-1.1±0.049	$<10^{-100}$	9,181	0.613±0.261	0.156	-1.22±0.0835	$<10^{-30}$	9,313	0.336±0.197	0.380	-1.05±0.0555	$<10^{-50}$
Arms fat mass	13,832	44.2±24.8	0.208	-106±7.38	$<10^{-30}$	6,863	37.6±38.2	0.444	-134.9±12.2	$<10^{-20}$	6,969	31.9±27.9	0.380	-93.5±7.83	$<10^{-30}$
Arms fat free mass	11,943	-12.2±26.1	0.669	-46.6±7.70	$<10^{-05}$	5,903	28±29.7	0.444	-52.5±9.4	$<10^{-05}$	6,040	-88.6±43.8	0.380	-26.6±12.2	0.0403
Arms lean mass	13,832	-12.0±23.4	0.669	-46±6.96	$<10^{-10}$	6,863	33.4±25.4	0.359	-48.5±8.13	$<10^{-05}$	6,969	-61.6±39.5	0.380	-27.2±11.1	0.0209
Arms tissue fat percentage	16,318	0.00322±0.00165	0.160	-0.00757±0.00049	$<10^{-50}$	8,113	0.00151±0.00242	0.556	-0.00951±0.000776	$<10^{-30}$	8,205	0.00306±0.00209	0.380	-0.00651±0.000581	$<10^{-20}$
Legs fat mass	13,832	90.6±64.4	0.285	-280.2±19.2	$<10^{-30}$	6,863	119±112	0.444	-360±35.8	$<10^{-20}$	6,969	92±73.2	0.380	-233±20.5	$<10^{-20}$
Legs fat free mass	13,832	-9.1±62.4	0.884	-112±18.6	$<10^{-05}$	6,863	83.2±82.1	0.444	-154±26.3	$<10^{-05}$	6,969	-107±97.1	0.380	-90.2±27.2	$<10^{-03}$
Legs lean mass	11,943	-34.4±74	0.669	-106±21.8	$<10^{-05}$	5,903	124±92.8	0.359	-148±29.4	$<10^{-05}$	6,040	-132±115	0.380	-85.4±32	0.0120
Legs tissue fat percentage	16,318	0.0022±0.00154	0.285	-0.00700±0.000456	$<10^{-50}$	8,113	0.00229±0.00245	0.444	-0.00782±0.000784	$<10^{-20}$	8,205	0.00217±0.00193	0.380	-0.00678±0.000538	$<10^{-30}$
Android fat mass	13,832	81.0±32.7	0.111	-196±9.73	$<10^{-50}$	6,863	104±48.5	0.198	-196±16	$<10^{-30}$	6,969	67.5±44.3	0.380	-194±12.42	$<10^{-50}$
Android fat free mass	11,943	19.7±14.8	0.288	-3.65±4.36	0.419	5,903	24.5±16.8	0.359	-11.5±5.31	0.0335	6,040	12.9±25.3	0.664	0.76±7.05	0.914
Android lean mass	13,832	15.5±13.2	0.334	-5.76±3.93	0.155	6,863	20.8±15.5	0.359	-10.5±4.97	0.0370	6,969	13.0±22.4	0.638	-2.49±6.28	0.720
Android tissue fat percentage	16,318	0.0037±0.0029	0.298	-0.0198±0.000861	$<10^{-100}$	8,113	0.00628±0.00478	0.359	-0.0221±0.00153	$<10^{-30}$	8,205	0.00368±0.00371	0.401	-0.0185±0.00103	$<10^{-50}$
Gynoid fat mass	13,832	63.3±36.7	0.212	-175±10.9	$<10^{-50}$	6,863	87.9±54.9	0.359	-206±17.6	$<10^{-30}$	6,969	53.9±45.6	0.380	-153±12.8	$<10^{-30}$
Gynoid fat free mass	11,943	99.2±29.2	0.0102	-17.78±8.61	0.0443	5,903	110±33.1	0.020	-36.3±10.5	$<10^{-03}$	6,040	42.9±47.2	0.432	-7.74±13.1	0.604
Gynoid lean mass	13,832	85.9±25.6	0.0102	-20.96±7.63	0.00715	6,863	91.5±28.9	0.020	-33.5±9.26	$<10^{-03}$	6,969	45.7±43.1	0.380	-9.11±12.1	0.512
Gynoid tissue fat percentage	16,318	0.00172±0.00155	0.351	-0.00933±0.000460	$<10^{-50}$	8,113	-0.000444±0.00247	0.858	-0.00929±0.000792	$<10^{-30}$	8,205	0.00323±0.00209	0.380	-0.00872±0.000583	$<10^{-30}$
Visceral adipose tissue mass	13,771	35.5±21.6	0.224	-121±6.42	$<10^{-50}$	6,825	21.3±23.9	0.444	-95.5±7.66	$<10^{-30}$	6,946	47.0±36.5	0.380	-152±10.2	$<10^{-30}$
Visceral adipose tissue volume	13,771	36.7±22.8	0.224	-128±6.79	$<10^{-50}$	6,825	22.8±25.4	0.444	-101±8.11	$<10^{-30}$	6,946	49.7±38.6	0.380	-161±10.8	$<10^{-30}$

**MRI**															
Total trunk fat volume	14,013	0.328±0.124	0.029	-0.780±0.0373	10^{-50}	6,980	0.067±0.186	0.720	-0.814±0.0607	$<10^{-30}$	7,033	0.429±0.165	0.022	-0.759±0.0461	$<10^{-50}$
Visceral adipose tissue volume	18,748	0.06±0.0470	0.281	-0.349±0.014	10^{-100}	9,309	0.0340±0.05	0.720	-0.257±0.0159	$<10^{-50}$	9,439	0.162±0.082	0.087	-0.464±0.0232	$<10^{-50}$
Abdominal subcutaneous adipose volume	18,744	0.149±0.0636	0.0447	-0.364±0.019	10^{-50}	9,308	0.128±0.107	0.720	-0.525±0.0342	$<10^{-50}$	9,436	0.109±0.072	0.182	-0.28±0.0202	$<10^{-30}$
Total adipose tissue volume	3,103	0.408±0.391	0.346	-1.189±0.119	10^{-20}	1,651	-0.483±0.576	0.720	-1.21±0.188	$<10^{-05}$	1,452	0.743±0.547	0.204	-1.071±0.155	$<10^{-10}$
Total lean tissue volume	3,103	0.098±0.170	0.562	0.0178±0.0514	0.730	1,651	0.248±0.188	0.720	0.0538±0.0611	0.378	1,452	-0.0946±0.256	0.712	-0.009±0.0729	0.898
Abdominal adipose tissue index (L/m^2)	14,013	0.135±0.0407	0.00661	-0.269±0.0122	10^{-100}	6,980	0.0354±0.068	0.720	-0.306±0.0222	$<10^{-40}$	7,033	0.158±0.05	0.0106	-0.245±0.0139	$<10^{-50}$
Thigh muscle fat infiltration percentage	13,738	0.0952±0.0435	0.0503	-0.189±0.0131	10^{-30}	6,940	0.0239±0.0629	0.720	-0.198±0.0205	$<10^{-20}$	6,798	0.165±0.0590	0.0170	-0.185±0.0164	$<10^{-30}$

Quantile (median) regression model (Model 2) includes age, sex, smoking status, Townsend deprivation level of recruiting center, education, household income, alcohol intake, and medication use (antihypertensive drugs, cholesterol lowering drugs, aspirin, and insulin). Subjects include participants with entries for all four online dietary recalls. Tissue masses are provided in kg, and tissue volumes are provided in L. Abdominal adipose tissue index is calculated as volume of adipose (L) divided by height squared (m^2). Correction for multiple comparisons performed with the Benjamini-Hochberg method.

Supplemental Table S9. Dietary manganese intake is associated with lipoprotein particle size, concentrations, and composition, as determined by NMR metabolomics for males and females separately.

**Lipid/lipoprotein Composition**	**Female only**				**Male only**			
	**rs13107325 (beta ± SE)**	**FDR rs13107325**	**Dietary Mn (beta ± SE)**	**FDR dietary Mn**	**rs13107325 (beta ± SE)**	**FDR rs13107325**	**Dietary Mn (beta ± SE)**	**FDR dietary Mn**
Average Diameter for HDL Particles	-0.0168±0.00388	$<10^{-03}$	0.0173±0.00127	$<10^{-30}$	-0.00843±0.00292	0.0186	0.00994±0.00082	$<10^{-30}$
Average Diameter for LDL Particles	-0.00132±0.00154	0.442	0.00348±0.000501	$<10^{-10}$	-0.0048±0.00176	0.0220	0.00351±0.000496	$<10^{-10}$
Average Diameter for VLDL Particles	0.0637±0.0221	0.00880	-0.0733±0.0072	$<10^{-20}$	0.0612±0.0241	0.0301	-0.0703±0.00678	$<10^{-20}$
Concentration of HDL Particles	-0.000095±0.0000405	0.0320	-0.0000576±0.0000132	$<10^{-03}$	-0.0000893±0.0000374	0.0385	-0.0000387±0.0000105	$<10^{-03}$
Concentration of Large HDL Particles	-0.0000758±0.0000145	$<10^{-05}$	0.000053±4.72E-06	$<10^{-20}$	-0.0000235±9.02E-06	0.0280	0.0000297±2.54E-06	$<10^{-30}$
Concentration of Medium HDL Particles	-0.0000485±0.0000152	0.00411	-6.55E-06±4.97E-06	0.194	-0.0000314±0.0000144	0.0530	-8.10E-06±4.06E-06	0.0485
Concentration of Small HDL Particles	-0.0000111±0.0000241	0.706	-0.000114±7.87E-06	$<10^{-40}$	-0.000021±0.0000231	0.456	-0.0000748±6.49E-06	$<10^{-20}$
Concentration of Very Large HDL Particles	-7.98E-06±1.73E-06	$<10^{-03}$	6.85E-06±5.65E-07	$<10^{-30}$	-2.11E-06±1.08E-06	0.0809	3.14E-06±3.03E-07	$<10^{-20}$
Triglycerides in Chylomicrons and Extremely Large VLDL	0.00292±0.00155	0.0854	-0.00394±0.000508	$<10^{-10}$	0.00614±0.00269	0.0426	-0.0099±0.0007572	$<10^{-30}$
Triglycerides in HDL	0.000342±0.000799	0.710	-0.00289±0.000261	$<10^{-20}$	0.000687±0.000854	0.512	-0.00257±0.0002401	$<10^{-20}$
Triglycerides in IDL	0.000513±0.000412	0.261	-0.00125±0.000135	$<10^{-15}$	0.000175±0.000477	0.758	-0.0017±0.000134	$<10^{-30}$
Triglycerides in LDL	0.00146±0.000583	0.0224	-0.00222±0.00019	$<10^{-30}$	0.000527±0.000726	0.530	-0.00287±0.000204	$<10^{-30}$
Triglycerides in Large HDL	-0.000257±0.000208	0.261	-0.00015±0.0000678	0.0291	-0.0000742±0.000202	0.7584	-0.000241±0.0000568	$<10^{-03}$
Triglycerides in Medium HDL	0.000325±0.000337	0.392	-0.00131±0.00011	$<10^{-30}$	0.000305±0.000333	0.456	-0.00107±0.0000938	$<10^{-20}$
Triglycerides in Small HDL	0.00113±0.000287	$<10^{-03}$	-0.00152±0.0000938	$<10^{-50}$	0.000458±0.000309	0.204	-0.00116±0.0000869	$<10^{-30}$
Triglycerides in VLDL	0.0137±0.00709	0.0834	-0.0255±0.00231	$<10^{-20}$	0.0127±0.0102	0.289	-0.03057±0.00287	$<10^{-20}$
Triglycerides in Very Large HDL	-0.0000583±0.0000412	0.213	-0.0000211±0.0000134	0.124	-8.95E-06±0.0000461	0.872	-0.0000738±0.000013	$<10^{-05}$
Triglycerides to Total Lipids in Large HDL percentage	0.0935±0.0337	0.0117	-0.144±0.011	$<10^{-30}$	0.193±0.0629	0.0145	-0.197±0.0177	$<10^{-20}$
Triglycerides to Total Lipids in Medium HDL percentage	0.0809±0.0314	0.0188	-0.116±0.0103	$<10^{-20}$	0.094±0.0339	0.0220	-0.0979±0.00952	$<10^{-20}$

Triglycerides to Total Lipids in Small HDL percentage	0.062±0.0211	0.00806	-0.076±0.0069	$<10^{-20}$	0.076±0.023	0.00928	-0.0612±0.00647	$<10^{-20}$
Triglycerides to Total Lipids in Very Large HDL percentage	0.1653±0.0327	$<10^{-05}$	-0.149±0.0107	$<10^{-30}$	0.147±0.0539	0.0220	-0.211±0.0152	$<10^{-30}$
Cholesterol in Large HDL	-0.0165±0.00325	$<10^{-05}$	0.0133±0.00106	$<10^{-30}$	-0.00637±0.00195	0.00928	0.00745±0.000548	$<10^{-30}$
Cholesterol in Medium HDL	-0.00575±0.00211	0.0126	0.000384±0.000687	0.578	-0.00437±0.00187	0.0397	0.00049±0.000527	0.363
Cholesterol in Small HDL	-0.000276±0.00113	0.806	-0.00477±0.000368	$<10^{-30}$	-0.000834±0.00108	0.514	-0.00298±0.000303	$<10^{-20}$
Cholesterol in Very Large HDL	-0.00313±0.000629	$<10^{-05}$	0.00283±0.000205	$<10^{-40}$	-0.000936±0.000374	0.0301	0.00133±0.000105	$<10^{-30}$
Cholesterol to Total Lipids in Large HDL percentage	-0.2641±0.0627	$<10^{-03}$	0.354±0.0205	$<10^{-50}$	-0.426±0.103	0.00117	0.442±0.0289	$<10^{-50}$
Cholesterol to Total Lipids in Medium HDL percentage	-0.138±0.0467	0.00806	0.181±0.0153	$<10^{-30}$	-0.155±0.0615	0.0301	0.173±0.0173	$<10^{-20}$
Cholesterol to Total Lipids Small HDL percentage	-0.0427±0.0318	0.234	0.0556±0.0104	$<10^{-05}$	-0.049±0.0372	0.271	0.0903±0.0105	$<10^{-15}$
Cholesterol to Total Lipids Very Large HDL percentage	0.103±0.0546	0.0854	-0.163±0.0178	$<10^{-15}$	0.145±0.0826	0.121	-0.162±0.0232	$<10^{-10}$
Total Lipids in HDL	-0.0458±0.0109	$<10^{-03}$	0.00992±0.00357	0.00624	-0.0268±0.00911	0.0183	0.00206±0.00256	0.422
Total Lipids in Large HDL	-0.0304±0.00597	$<10^{-05}$	0.0217±0.00195	$<10^{-20}$	-0.0128±0.00378	0.00928	0.0118±0.00106	$<10^{-20}$
Total Lipids in Medium HDL	-0.00873±0.0038	0.0348	-0.00362±0.00124	0.00413	-0.0074±0.00347	0.0559	-0.00265±0.000975	0.00718
Total Lipids in Small HDL	0.00109±0.00279	0.716	-0.0142±0.000898	$<10^{-50}$	-0.000317±0.00279	0.910	-0.0100±0.000785	$<10^{-30}$
Total Lipids in Very Large HDL	-0.00759±0.00148	$<10^{-05}$	0.0063±0.000482	$<10^{-30}$	-0.00202±0.000862	0.0397	0.00304±0.000242	$<10^{-30}$

Quantile (median) regression models (Model 2 used for all) include age, sex, Townsend deprivation level of recruiting center, education, household income, smoking status, alcohol intake frequency, medication use (antihypertensive medications, cholesterol-lowering medications, aspirin, and insulin), physical activity, dietary energy intake, and fasting time. Dietary manganese includes subjects with at least one in-person or online dietary entry for dietary manganese and energy intake. N= 31,042 for women only, except for triglycerides to total lipids in very large HDL percentage (N= 31,034). N= 28,621 for men only, except for triglycerides to

total lipids in very large HDL percentage (N= 28,600). Correction for multiple comparisons performed with the Benjamini-Hochberg method.

Supplemental Table S10. Dietary manganese intake is associated with lipoprotein particle size, concentrations, and composition, as determined by NMR metabolomics, in UK Biobank participants with all four online dietary recalls.

Lipid/lipoprotein Composition	Both sexes				Female only				Male only			
	**rs13107325 (beta ± SE)**	**FDR rs13107325**	**Dietary Mn (beta ± SE)**	**p-values dietary Mn**	**rs13107325 (beta ± SE)**	**FDR rs13107325**	**Dietary Mn (beta ± SE)**	**FDR dietary Mn**	**rs13107325 (beta ± SE)**	**FDR rs13107325**	**Dietary Mn (beta ± SE)**	**FDR dietary Mn**
Average Diameter for HDL Particles	-0.00245±0.00664	0.734	0.0196±0.00223	$<10^{-15}$	0.00596±0.0111	0.816	0.0220±0.00400	$<10^{-05}$	-0.0111±0.00780	0.400	0.0155±0.00247	$<10^{-05}$
Average Diameter for LDL Particles	-0.00156±0.00324	0.690	0.00576±0.00109	$<10^{-05}$	0.00233±0.00417	0.816	0.00579±0.00149	$<10^{-03}$	-0.01±0.00501	0.388	0.00489±0.00160	0.00255
Average Diameter for VLDL Particles	0.04079±0.04526	0.540	-0.108±0.0152	$<10^{-10}$	0.0340±0.0597	0.816	-0.1139±0.0214	$<10^{-05}$	0.0622±0.0689	0.533	-0.106±0.0218	$<10^{-05}$
Concentration of HDL Particles	-0.000144±0.0000733	0.322	-0.0000783±0.0000246	0.00179	-0.0000525±0.000106	0.816	-0.0000945±0.0000380	0.0130	-0.0002227±0.0000973	0.376	-0.0000555±0.0000308	0.0860
Concentration of Large HDL Particles	-0.0000224±0.0000248	0.540	0.000055±8.32E-06	$<10^{-10}$	0.0000287±0.0000423	0.816	0.0000791±0.0000152	$<10^{-05}$	-0.0000333±0.0000254	0.432	0.0000481±8.06E-06	$<10^{-05}$
Concentration of Medium HDL Particles	-0.0000229±0.0000279	0.540	-0.0000112±9.38E-06	0.241	-5.30E-06±0.0000416	0.986	-0.0000118±0.0000149	0.428	-0.0000537±0.0000386	0.400	-6.29E-06±0.0000122	0.607
Concentration of Small HDL Particles	-0.0000446±0.000042	0.540	-0.000132±0.0000141	$<10^{-10}$	-0.0000348±0.0000678	0.816	-0.000154±0.0000243	$<10^{-05}$	8.50E-06±0.0000615	0.890	-0.000114±0.0000195	$<10^{-05}$
Concentration of Very Large HDL Particles	-3.03E-06±2.87E-06	0.540	7.63E-06±9.65E-07	$<10^{-10}$	-2.26E-06±4.86E-06	0.816	0.0000107±0.00000174	$<10^{-05}$	3.27E-06±2.87E-06	0.467	6.12E-06±9.09E-07	$<10^{-05}$
Triglycerides in Chylomicrons and Extremely Large VLDL	0.0071±0.00411	0.343	-0.00744±0.00138	$<10^{-05}$	0.00202±0.00411	0.816	-0.00611±0.00148	$<10^{-03}$	0.00872±0.00758	0.467	-0.0117±0.0024	$<10^{-05}$
Triglycerides in HDL	0.00207±0.0015	0.472	-0.00327±0.000502	$<10^{-05}$	0.000894±0.00228	0.842	-0.00336±0.000816	$<10^{-03}$	0.00324±0.00214	0.400	-0.00329±0.000678	$<10^{-05}$
Triglycerides in IDL	0.000349±0.000813	0.709	-0.00168±0.000273	$<10^{-05}$	0.000506±0.00111	0.816	-0.00127±0.000397	0.00135	0.00034±0.00122	0.837	-0.00227±0.000386	$<10^{-05}$
Triglycerides in LDL	0.000767±0.00121	0.616	-0.00315±0.000406	$<10^{-10}$	0.00152±0.00152	0.816	-0.00259±0.000544	$<10^{-05}$	0.00101±0.00196	0.764	-0.00382±0.00062	$<10^{-05}$
Triglycerides in Large HDL	0.000968±0.000413	0.315	-0.000231±0.000139	0.105	0.00106±0.000586	0.658	-0.000117±0.00021	0.579	0.000455±0.00057	0.577	-0.000323±0.000181	0.0860
Triglycerides in Medium HDL	0.000764±0.000642	0.540	-0.00139±0.000216	$<10^{-05}$	0.0000646±0.000913	0.986	-0.00154±0.000327	$<10^{-05}$	0.000993±0.000883	0.467	-0.00139±0.00028	$<10^{-05}$
Triglycerides in Small HDL	0.00104±0.000613	0.343	-0.00172±0.000206	$<10^{-15}$	0.00132±0.000842	0.658	-0.00187±0.000302	$<10^{-05}$	0.000834±0.000846	0.533	-0.00156±0.000268	$<10^{-05}$
Triglycerides in VLDL	0.0136±0.0161	0.540	-0.0368±0.00541	$<10^{-10}$	0.0124±0.0199	0.816	-0.0332±0.00712	$<10^{-05}$	0.0116±0.0267	0.807	-0.0394±0.00847	$<10^{-05}$
Triglycerides in Very Large HDL	0.0000751±0.0000869	0.540	-0.0000526±0.0000292	0.084	0.000211±0.000118	0.658	-0.0000461±0.0000423	0.275	0.0000234±0.000127	0.879	-0.0000643±0.0000401	0.123
Triglycerides to Total Lipids in Large HDL percentage	0.138±0.0882	0.399	-0.228±0.0296	$<10^{-10}$	0.0973±0.0924	0.816	-0.153±0.0331	$<10^{-05}$	0.285±0.171	0.400	-0.282±0.0541	$<10^{-05}$
Triglycerides to Total Lipids in Medium HDL percentage	0.139±0.0643	0.315	-0.148±0.0216	$<10^{-10}$	0.143±0.0869	0.658	-0.145±0.0312	$<10^{-05}$	0.167±0.096	0.400	-0.133±0.0303	$<10^{-03}$

Triglycerides to Total Lipids in Small HDL percentage	0.0896±0.0430	0.315	-0.0986±0.0144	$<10^{-15}$	0.0954±0.058	0.658	-0.0966±0.0208	$<10^{-05}$	0.102±0.06	0.400	-0.0858±0.0190	$<10^{-05}$
Triglycerides to Total Lipids in Very Large HDL percentage	0.0859±0.0814	0.540	-0.232±0.0274	$<10^{-15}$	0.0626±0.092	0.816	-0.161±0.033	$<10^{-05}$	0.148±0.152	0.533	-0.310±0.0481	$<10^{-05}$
Cholesterol in Large HDL	-0.00581±0.00549	0.540	0.0131±0.00184	$<10^{-10}$	0.00512±0.00924	0.816	0.0184±0.00331	$<10^{-05}$	-0.00882±0.00593	0.400	0.0110±0.00188	$<10^{-05}$
Cholesterol in Medium HDL	-0.00425±0.00382	0.540	0.000545±0.00128	0.672	0.00347±0.00532	0.816	-0.00113±0.00191	0.554	-0.00607±0.00517	0.467	0.00210±0.00164	0.218
Cholesterol in Small HDL	-0.00176±0.00196	0.540	-0.00547±0.000657	$<10^{-15}$	-0.00189±0.00322	0.816	-0.0066±0.00115	$<10^{-05}$	-0.0027±0.00291	0.533	-0.00410±0.000922	$<10^{-03}$
Cholesterol in Very Large HDL	-0.000838±0.00102	0.540	0.00295±0.000343	$<10^{-15}$	-0.00137±0.00178	0.816	0.00396±0.00064	$<10^{-05}$	-0.00062±0.00105	0.724	0.00210±0.000333	$<10^{-10}$
Cholesterol to Total Lipids in Large HDL percentage	-0.339±0.158	0.315	0.532±0.0530	$<10^{-20}$	-0.151±0.172	0.816	0.4348±0.0617	$<10^{-10}$	-0.613±0.261	0.376	0.630±0.0828	$<10^{-10}$
Cholesterol to Total Lipids in Medium HDL percentage	-0.191±0.100	0.322	0.244±0.0336	$<10^{-10}$	-0.149±0.127	0.816	0.2449±0.0454	$<10^{-05}$	-0.322±0.16	0.388	0.231±0.0507	$<10^{-05}$
Cholesterol to Total Lipids Small HDL percentage	-0.11±0.0630	0.343	0.095±0.0212	$<10^{-05}$	-0.0404±0.0873	0.816	0.0844±0.0313	0.00706	-0.137±0.097	0.400	0.105±0.0308	$<10^{-03}$
Cholesterol to Total Lipids Very Large HDL percentage	-0.195±0.130	0.416	-0.263±0.0437	$<10^{-05}$	-0.271±0.149	0.658	-0.218±0.0534	$<10^{-03}$	-0.0874±0.241	0.837	-0.338±0.0764	$<10^{-03}$
Total Lipids in HDL	-0.0173±0.0195	0.540	0.00779±0.00655	0.241	0.00952±0.029	0.842	0.0144±0.0104	0.166	-0.0354±0.0252	0.400	0.00942±0.00797	0.252
Total Lipids in Large HDL	-0.00734±0.0106	0.615	0.02322±0.00356	$<10^{-05}$	0.0157±0.0173	0.816	0.0323±0.00621	$<10^{-05}$	-0.0178±0.011	0.400	0.0187±0.00348	$<10^{-05}$
Total Lipids in Medium HDL	-0.00469±0.00709	0.616	-0.00402±0.00238	0.103	-0.000512±0.0105	0.986	-0.00493±0.00375	0.214	-0.00831±0.00939	0.533	-0.00309±0.00298	0.307
Total Lipids in Small HDL	0.000992±0.00548	0.856	-0.0152±0.00184	$<10^{-15}$	0.00253±0.00767	0.842	-0.0167±0.00275	$<10^{-05}$	0.00211±0.00781	0.837	-0.0146±0.00247	$<10^{-05}$
Total Lipids in Very Large HDL	-0.00148±0.00247	0.622	0.00688±0.00083	$<10^{-15}$	-0.000076±0.00421	0.986	0.00924±0.00151	$<10^{-05}$	-0.000717±0.0024	0.837	0.00568±0.000761	$<10^{-10}$

Quantile (median) regression model (Model 2) includes age, sex, smoking status, Townsend deprivation level of recruiting center, education, household income, fasting time, alcohol intake, and medication use (antihypertensive drugs, cholesterol lowering drugs, aspirin, and insulin). Dietary manganese includes subjects with all four online dietary entries for dietary manganese and energy intake. N=8,051 for both sexes, except for triglycerides to total lipids in very large HDL percentage (N= 8,045). N=4,282 for women only, except for triglycerides to total lipids in very large HDL percentage (N= 4,279). N= 3,769 for men only, except for triglycerides

to total lipids in very large HDL percentage (N= 3,766). Correction for multiple comparisons performed with the Benjamini-Hochberg method
